# DNA conformational transitions inferred from re-evaluation of *m*|*F*_o_| − *D*|*F*_c_| electron-density maps

**DOI:** 10.1107/S2059798317007707

**Published:** 2017-06-22

**Authors:** Tomoko Sunami, Toshiyuki Chatake, Hidetoshi Kono

**Affiliations:** a Molecular Modeling and Simulation Group, National Institutes for Quantum and Radiological Science and Technology, 8-1-7 Umemidai, Kizugawa 619-0215, Japan; b Research Reactor Institute, Kyoto University, 2 Asashironishi, Kumatori, Osaka 590-0494, Japan

**Keywords:** crystallographic heterogeneity, DNA, flexibility of phosphate backbone, Z-DNA, ZI/ZII transition

## Abstract

Conformational flexibility of DNA plays important roles in biological processes such as transcriptional regulation and DNA packaging *etc*. To understand the mechanisms of these processes, it is important to analyse when, where and how DNA shows conformational variations. Recent analyses have indicated that conventional refinement methods do not always provide accurate models of crystallographic heterogeneities and that some information on polymorphism has been overlooked in previous crystallographic studies. In the present study, the *m*|*F*_o_| − *D*|*F*_c_| electron-density maps of double-helical DNA crystal structures were calculated at a resolution equal to or better than 1.5 Å and potential conformational transitions were found in 27% of DNA phosphates. Detailed analyses of the *m*|*F*_o_| − *D*|*F*_c_| peaks indicated that some of these unassigned densities correspond to ZI ↔ ZII or A/B → BI conformational transitions. A relationship was also found between ZI/ZII transitions and metal coordination in Z-DNA from the detected peaks. The present study highlights that frequent transitions of phosphate backbones occur even in crystals and that some of these transitions are affected by the local molecular environment.

## Abstract 2

A re-evaluation of *m*|*F*_o_| − *D*|*F*_c_| electron-density maps revealed that potential conformational transitions of 27% of DNA phosphates are found in previous crystallographic data. The analysis suggests that some of these unassigned densities correspond to ZI ↔ ZII or A/B → BI transitions.

## 1. Introduction   

DNA plasticity plays important roles in various biological processes such as the transcription and packaging of genomic information. A representative example of such plasticity is the sequence-dependent deformability of DNA, which has been shown to be crucial for molecular recognition of DNA-binding proteins (Olson *et al.*, 1998 ▸; Sarai & Kono, 2005 ▸; Rohs *et al.*, 2009 ▸). B-DNA adopts two representative phosphate backbone conformations, BI and BII, which are defined by ∊–ζ values (Supplementary Fig. S1). BI is known to be a major conformer in B-DNA (Schneider *et al.*, 1997 ▸). Several studies have been performed to analyse the DNA deformability from this aspect, as exemplified in the following. NMR data revealed evidence for a BI–BII equilibrium in steps flanking the NF-κB binding site and a curvature of the DNA duplex induced by the equilibrium (Tisné *et al.*, 1998 ▸; Wecker *et al.*, 2002 ▸). A mutation study showed that the distribution of BI–BII conformational equilibria had a critical effect on the binding affinity (Tisné *et al.*, 1999 ▸). Molecular-dynamics (MD) simulations of the binding sites of papillomavirus type 1 E2 protein (Djuranovic & Hartmann, 2005 ▸; Robertson & Cheat­ham, 2015 ▸) demonstrated that a DNA sequence with stronger affinity adopts the BII conformer more frequently, and the BII conformer is also preserved in protein–DNA complexes. In this way, transitions between BI and BII have mainly been studied using MD or NMR spectroscopy.

In contrast, only a few studies have been performed to analyse DNA flexibility using crystallography. In these studies, the *B* factor, which is obtained by crystallographic analysis, has mainly been used. This parameter related to atomic thermal motion is expressed as a probability density function of atomic displacements from the mean position. It has been reported that tetra-acetylation of H4 histones significantly increases the *B* factors of wrapped DNAs in the vicinity of the tetra-acetylated N-terminal tail of inter- and intra-nucleosomal H4 (Wakamori *et al.*, 2015 ▸). From this observation and biochemical assays, it is suggested that H4 tetra-acetylation impairs the self-association of nucleosome core particles by changing the interactions of the H4 tail with DNA. Schneider *et al.* (2014 ▸) analysed the distributions of scaled *B* factors from the crystal structures of protein–DNA complexes using a bioinformatic approach. They found that DNA backbones have large *B*-factor values even when complexed with proteins, but the distributions of the *B* factors of free DNA backbones are so extremely wide that they are comparable to those of surface waters. However, it is difficult to capture large conformational changes such as phosphate backbone transitions using *B* factors, assuming that the distribution of atomic positions in the crystal may only have a spherical or elliptical pattern in the *B*-factor calculation.

To deal with the variety of molecular conformations in crystals, multiple conformations have been assigned to part of the molecule. Polymorphisms of phosphate backbones have sometimes been identified as multiple conformations (Wang *et al.*, 1981 ▸; Egli *et al.*, 1998 ▸; Kielkopf *et al.*, 2000 ▸). These identifications are usually limited to high-resolution crystal structures. Ensemble refinements, which are novel structural refinement methods, have been proposed to describe structural variations precisely rather than just assigning multiple conformations to part of the molecule (DePristo *et al.*, 2004 ▸; Levin *et al.*, 2007 ▸). In these methods, multiple copies of initial models are refined at the same time. The methods generally improve refinement statistics such as *R*_free_. However, which kind of ensemble refinement most precisely describes the heterogeneity of the molecular structure and how to distinguish crystallographic heterogeneity from uncertainty of the refined model remain controversial (Terwilliger *et al.*, 2007 ▸; Knight *et al.*, 2008 ▸; Burnley *et al.*, 2012 ▸). Recently, Lang and coworkers have developed a program which takes samples of electron density around the dihedral angles of protein side chains (Lang *et al.*, 2010 ▸). They revealed that densities less than 1σ but higher than that of an H atom are significantly enriched in 2|*F*_o_| − |*F*_c_| maps obtained by conventional refinement. These densities correspond to minor rotational conformations. Using these weak densities, they observed a change in the distribution of conformations of side chains on cooling crystals (Fraser *et al.*, 2011 ▸). Such observations also revealed a low-occupancy location of an inhibitor of HIV capsid protein and a change of conformation ensembles in the allosteric network of protein kinases upon substrate binding (Lang *et al.*, 2014 ▸). These studies indicated that structural information about minor conformations may still be obtained from electron-density maps derived from conventional X-ray refinement. As far as we know, these analyses have only been applied to protein side chains.

In the present study, a comprehensive analysis of electron-density maps of DNA crystal structures was carried out in order to discover unassigned conformational transitions in these maps. The *m*|*F*_o_| − *D*|*F*_c_| electron-density map was used, which represents differences between structure factors derived from experimental X-ray diffractions and those calculated by a Fourier transform of the model coordinates (Henderson & Moffat, 1971 ▸; Read, 1986 ▸). Therefore, the map is expected to provide us with information about crystallo­graphic heterogeneities that were neglected in the refined models. The *m*|*F*_o_| − *D*|*F*_c_| maps of DNA crystal structures which were solved at a resolution equal to or better than 1.5 Å were recalculated. Peaks were picked out from the maps to identify the locations of potential alternative conformations. The peak frequency around each DNA atom was calculated. How the frequency of appearance of the peak is affected by resolution and DNA local structure was also evaluated. As a result, we found that more peaks around P atoms appear than we expected, which indicates frequent transitions of phosphate backbones, and that better resolution data have more peaks. Furthermore, we inferred the transitions that these peaks correspond to. Finally, it is demonstrated that local environments such as metal coordination affect the transitions.

## 2. Materials and methods   

### 2.1. Data-set extraction from the PDB, refinement of *B* factors and peak search   

153 sets of coordinates and X-ray structure factors of DNA structures which were solved at a resolution equal to or better than 1.5 Å were extracted from the Protein Data Bank (PDB; November 2014). Before the calculation of density maps, individual *B* factors were refined using the *PHENIX* software suite (Adams *et al.*, 2010 ▸). 5% of the total reflections were excluded from refinement for the calculation of *R*_free_. Finally, 147 sets of refined models and structure factors were obtained, after discarding six structures for which the *R* factors were too high (*R*_work_ ≥ 0.27 or *R*_free_ ≥ 0.30) or for which topology files could not be automatically generated by* phenix.elbow*. According to NDB classification, they consist of 39 A-DNA structures, 61 B-DNA structures, 22 Z-DNA structures and 25 others.

*m*|*F*_o_| − *D*|*F*_c_| maps were then generated at 1.5 Å resolution. Peaks were picked from the asymmetric unit of the *m*|*F*_o_| − *D*|*F*_c_| maps using *PEAKMAX* in the *CCP*4 software suite (Winn *et al.*, 2011 ▸). The threshold of the peak intensity was set to 3.5σ. The threshold chosen is further discussed in §[Sec sec3.1]3.1. The peaks were relocated in the vicinity of DNA molecules using the *SORTWATER* module from the *CCP*4 software suite. To evaluate how often peaks appear around the atoms of interest, we calculated the frequency *f*_p_, which is defined as 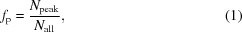
where *N*_all_ is the total number of atoms and *N*_peak_ is the number of atoms which contain one or more peaks within 2.2 Å of the atom.

In order to analyse the effect of X-ray resolution, *m*|*F*_o_| − *D*|*F*_c_| electron-density maps were generated at five different resolutions as follows. The same X-ray refinements as those in the above procedure were performed for DNA crystal structures solved at a resolution equal to or better than 2.5 Å. The *m*|*F*_o_| − *D*|*F*_c_| map was calculated at 1.0, 1.25, 1.5, 1.9 and 2.5 Å resolution for data of ≤1.0, 1.0–1.25, 1.25–1.5, 1.5–1.9 and 1.9–2.5 Å resolution, respectively. A search was made for peaks in the maps in the same way as above.

In this study, the r.m.s. of densities (σ) was used to distinguish peaks from noise. The density of electrons (e Å^−3^) can also be used and this different measure might give a different result. We then investigated the variation of electron density corresponding to 1σ in the electron-density maps. Supplementary Fig. S2 shows the distribution of the electron densities of the structures. The values for each structure are listed in Supplementary Table S1. Most of the structures in our data set have similar electron-density values, while some of them have higher values. To examine the contribution of these outliers to the result, we analysed data excluding 25% of the structures with the highest or lowest electron density/σ. We observed no significant differences compared with the case using all data (data not shown). Therefore, we use σ as the measure in this study.

We also considered the effect of Fourier truncation in the data analysis. Theoretically, Fourier truncation generates ripples. For instance, a ripple can appear at a distance of 2.2 Å around a strong peak in a 1.5 Å resolution map. We checked the 15 strongest peaks in our data set and confirmed that no ring-like densities appeared around the strong peaks in the maps, which indicates that the effect of the truncation is negligibly small when a 3.5σ cutoff is used.

PDB codes, resolutions, *R*_work_, *R*_free_ and NDB classifications are listed in Supplementary Table S2.

### 2.2. Classification of conformations of dinucleotides   

We classified the conformations of each dinucleotide in the data set. The classification was used to anticipate possible transitions corresponding to the peaks in *m*|*F*_o_| − *D*|*F*_c_| maps. In order to select DNA double-stranded structures only, DNA regions flipped-out from the duplexes, DNA intercalator-binding sites and ssDNA were excluded by visual inspection. Dinucleotides which contain bases other than DA, DT, DG and DC were also excluded (the naming rules for residues used in the PDB were followed in this paper).

The classification into AI, AII, BI, BII, A/B, B/A, ZI (puRine–pYrimidine steps; hereafter R–Y steps), ZII (R–Y steps) or Z (Y–R steps) was performed according to the distances to nine conformer clusters created by Svozil *et al.* (2008 ▸). The torsion angles δ, ∊, ζ, α1, β1, γ1 and δ1 calculated using *X*3*DNA* (Lu & Olson, 2003 ▸) were used to calculate the Euclidean distance to the cluster centres. If the distance to any of them was larger than 35°, those dinucleotides were excluded from further analyses. As a result, 93% of A-DNA, 96% of B-DNA, 95% of Z-DNA (R–Y steps) and 71% of Z-DNA (Y–R steps) were assigned to one of these nine conformers.

### 2.3. Simulation data preparation   

In order to determine a borderline between noise and signal in the *m*|*F*_o_| − *D*|*F*_c_| maps, a simulation with ||*F*_o_| − |*F*_c_|| (hereafter called FoFc) error or _refln.F_meas_sigma_au in the deposited structure factors (sigmaF) error was performed. In general, *m*|*F*_o_| − *D*|*F*_c_| maps include errors generated from a combination of the errors from the diffraction data and from incomplete modelling. Thus, FoFc includes both. SigmaF includes only the measurement error. The errors were assumed to be distributed in a Gaussian form. A similar assumption was previously adopted to calculate a RAPID map (Lang *et al.*, 2014 ▸), which estimates the noise in 2*m*|*F*_o_| − *D*|*F*_c_| maps of protein structures.

The procedure for the simulations involves the following steps (see the flowchart for the procedure in Supplementary Fig. S3): (i) calculating |*F*_calc_| for representative DNA structures; (ii) introducing random noise to |*F*_calc_| in order to prepare substitutes for experimental structure factors (hereafter referred to as |*F*′_obs_|); (iii) refining the models using |*F*′_obs_|; and (iv) picking peaks from the *m*|*F*′_obs_| − *D*|*F*′_calc_| maps [|*F*′_calc_| are the calculated structure factors obtained from (iii)].

Details of steps of (i)–(iv) are described below.

(i) In the simulation, the structures with the best resolution were chosen from A-DNA, B-DNA and Z-DNA: PDB entries 1dpl, 1d8g and 3p4j, respectively (Egli *et al.*, 1998 ▸; Kielkopf *et al.*, 2000 ▸; Brzezinski *et al.*, 2011 ▸). |*F*_calc_| was obtained using *SFALL* from *CCP*4 after running ten cycles of refinement using *PHENIX*. The refinement parameters were individual atomic coordinates, individual *B* factors and occupancy for multiple locations.
(ii) |*F*′_obs_| was calculated by adding a Gaussian noise to |*F*_calc_|,
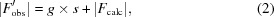
where *g* is a random sample of Gaussian distribution with a mean of zero and standard deviation of 1 and *s* is FoFc or sigmaF. The former is directly related to the error in *m*|*F*_o_| − *D*|*F*_c_| maps. The latter only includes the measurement error in the diffraction experiment.
(iii) For 1.0–1.9 Å resolution data, ten cycles of refinement were run using |*F*′_obs_|. The initial models were the coordinates obtained in (i). Water molecules were then removed from the refined structures. An additional ten cycles of refinement were performed with the ordered_solvent option, which automatically adds water molecules. The refinement parameters were the individual atomic coordinates, individual *B* factors and occupancy for multiple locations in both refinement steps. For 2.5 Å resolution data, ten cycles of rigid-body refinement were carried out with the ordered_solvent option. The initial models were the coordinates obtained from the first refinement of 1.9 Å resolution data.
(iv) Peaks were calculated using *PEAKMAX* and *SORTWATER* using the same procedure as that used to obtain those for the experimental data shown above.

To obtain the statistics of peak frequencies, steps (i)–(iv) were repeated 100 times with different random seed values.

The *pandas* software library (http://pandas.pydata.org/) was used for statistical analyses and the mmLib library for the superimpositions of dinucleotides (Painter & Merritt, 2004 ▸). All molecular structures in the present paper were drawn using *PyMOL* (v.1.8; Schrödinger).

## 3. Results   

### 3.1. Determination of a threshold for peak picking in *m*|*F*_o_| − *D*|*F*_c_| maps   

In order to determine a threshold for reliably picking peaks in the *m*|*F*_o_| − *D*|*F*_c_| maps, we first estimated the frequency of peaks in the *m*|*F*_o_| − *D*|*F*_c_| maps that can be produced by noise from the errors in the measurement and modelling procedure. We carried out a simulation of *m*|*F*_o_| − *D*|*F*_c_| maps of the three representative DNA structures at 1.5 Å resolution, adding random noise to the structure factors. In the simulation, two types of error were considered as a source of noise: FoFc and sigmaF. FoFc includes the measurement error and the error from incomplete modelling and is directly related to the noise in *m*|*F*_o_| − *D*|*F*_c_| maps. SigmaF only reflects the measurement error in the diffraction experiment.

**Figure 1:**
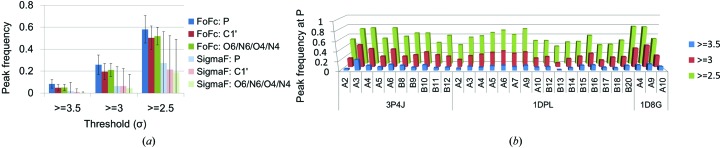
Peak frequencies which can be produced by noise in the *m*|*F*_o_| − *D*|*F*_c_| maps from experimental and modelling errors. (*a*) Peak frequencies around P, C1′ and O6/N6/O4/N4 atoms (the sixth heteroatom in purine and the fourth heteroatom in pyrimidine) are shown at thresholds of 3.5, 3 or 2.5σ. Simulations were carried out with FoFc error or sigmaF error. The bar indicates standard deviation within all residues. (*b*) Peak frequencies of P atoms in the simulation with FoFc error. Atoms without alternative locations were used to draw the figures.

Based on the simulated maps with FoFc error, the threshold of σ to pick peaks in the experimental data set was determined as follows. Frequencies of peaks which appeared at a distance of within 2.2 Å of the P atoms of phosphates, C1′ of sugars and the sixth heteroatom in purine and the fourth heteroatom in pyrimidine were calculated in the *m*|*F*_o_| − *D*|*F*_c_| maps at 1.5 Å resolution (Fig. 1 ▸ and Supplementary Fig. S4*a*). The distance of 2.2 Å is discussed in §[Sec sec3.2]3.2. At a threshold level of 3.5σ, the frequency of peaks produced by noise was low at about 5%. In contrast, at threshold levels of 3.0σ or 2.5σ the peaks were found with higher probabilities of about 25 or 60%, respectively. There were no significant differences among the peak frequencies for different atom types or different models. On the other hand, the average level of noise using sigmaF was relatively low (Fig. 1 ▸ *a* and Supplementary Fig. S4*b*). This is reasonable because in general a large gap between *R*_merge_ (corresponding to sigmaF) and the *R* factor in the refinement process (corresponding to FoFc) exists. This gap has been reported to come from isotropic *B* factors and coordinate error (Vitkup *et al.*, 2002 ▸). We thus decided to use 3.5σ as a threshold for the peak search in our analyses.

### 3.2. Distribution of peaks in *m*|*F*_o_| − *D*|*F*_c_| maps   

**Figure 2:**
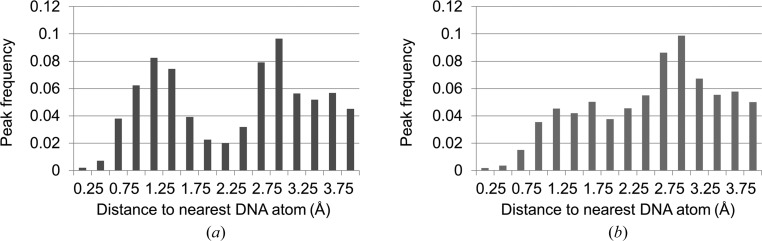
Distances between peaks and their nearest DNA atoms. The distances between peaks and their nearest DNA atoms were calculated using peaks derived from (*a*) the experimental *m*|*F*_o_| − *D*|*F*_c_| maps and (*b*) the simulated *m*|*F*_o_| − *D*|*F*_c_| maps.

Peaks with a threshold of 3.5σ were extracted from *m*|*F*_o_| − *D*|*F*_c_| maps which were experimentally obtained as well as from those that were simulated. The distribution of distances between the peaks and their nearest DNA atom was plotted as shown in Fig. 2 ▸. In the *m*|*F*_o_| − *D*|*F*_c_| maps from experimental data, peaks were often observed around distances of 1.25 and 3.0 Å from DNA (Fig. 2 ▸ *a*). Peaks around 3.0 Å were also found in the simulated *m*|*F*_o_| − *D*|*F*_c_| maps (Fig. 2 ▸ *b*). In the simulated maps, many peaks around 3.0 Å appeared in the region corresponding to water molecules. Although some such water molecules were reassigned in the simulation, there were a certain number of these that were not assigned. Most peaks around 3.0 Å from DNA in the experimental data sets may be considered to correspond to unassigned solvent atoms as well, because low-occupancy solvent atoms might not be possible to be assigned in the refinement procedure. Only in the experimental data set were peaks found around 1.25 Å. It may be inferred that these peaks are derived from the movement of DNA itself, because the distances from DNA are too short to be interpreted as coming from densities of other atoms. In addition, the distances are too long to be interpreted as a part of thermal vibration of the atoms around mean positions, because the root-mean-square deviation of atom displacement was estimated to be 0.4 Å from a *B* factor of 15 Å^2^. Therefore, it was concluded that the peaks correspond to conformational transitions rather than thermal vibrations of individual atoms.

**Figure 3:**
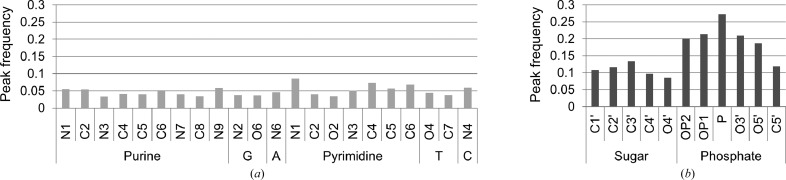
Peak frequencies of DNA atoms (*a*) in bases and (*b*) in sugars and phosphates. Atoms without alternative locations were used to draw the figures.

Next, the peak frequencies of atoms of DNA were analysed. To calculate peak frequencies, we considered peaks at distances within 2.2 Å of DNA atoms, because the peak distribution has a maximum around 1.25 Å, after which it decreases and finally reaches a minimum around 2.2 Å (Fig. 2 ▸ *a*). Therefore, it is assumed that peaks which appear at a distance shorter than 2.2 Å cover most conformational transitions of DNA. The calculated frequencies are shown in Fig. 3 ▸. The frequencies of peaks are about 0.05 for any atoms of bases. They are almost the same as or a little lower than the noise levels derived from the simulation, as shown in the previous section. In contrast, the peak frequencies for the atoms in sugar and phosphate are higher at 0.08–0.27. P atoms, in particular, most often accompany *m*|*F*_o_| − *D*|*F*_c_| densities. The scattering factor of P is about twice as large as those of C, O and N. This is probably why alternative locations of P atoms are more often detected than those of other atoms. As shown using NMR and MD, phosphate backbones equilibrate with different conformations in solution (Tisné *et al.*, 1998 ▸; Wecker *et al.*, 2002 ▸; Djuranovic & Hartmann, 2005 ▸; Robertson & Cheatham, 2015 ▸). Thus, it was interpreted that the peaks found here most probably correspond to different backbone conformations. In summary, the analyses in this section reveal that the experimental data contains structural information related to transitions of the phosphate backbones. The transitions can be traced by examining the electron density around P atoms in the *m*|*F*_o_| − *D*|*F*_c_| maps.

### 3.3. Resolution dependence   

Next, the effects of the X-ray resolution on the electron-density maps were examined. Data sets containing crystal structures at or better than 2.5 Å resolution were divided into five bins (*d* ≤ 1.0, 1.0 < *d* ≤ 1.25, 1.25 < *d* ≤ 1.5, 1.5 < *d* ≤ 1.9 and 1.9 < *d* ≤ 2.5 Å, where *d* denotes the resolution) according to their resolution. The *m*|*F*_o_| − *D*|*F*_c_| maps were calculated at the upper resolution limit of each bin.

**Figure 4:**
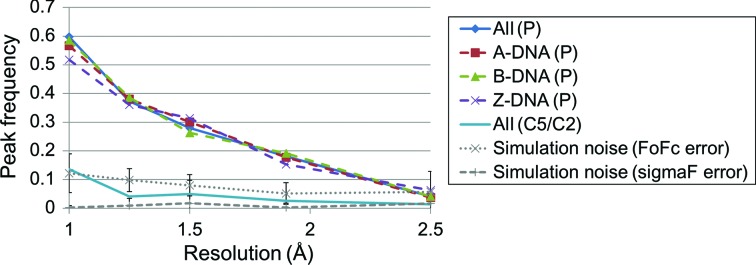
Resolution dependence of peak frequencies in *m*|*F*_o_| − *D*|*F*_c_| maps. The *m*|*F*_o_| − *D*|*F*_c_| maps were drawn at the resolution of the upper limit of each bin. ALL (P), A-DNA (P), B-DNA (P) and Z-­DNA (P) denote the peak frequencies of P atoms in all DNA forms, in A-DNA, in B-DNA and in Z-­DNA, respectively. ALL (C5/C2) is the peak frequency of C5 of purine or C2 of pyrimidine in all DNA forms. The peak frequencies in *m*|*F*_o_| − *D*|*F*_c_| maps obtained by simulations using FoFc error or sigmaF error are also shown by a dotted line with cross marks and a dashed line with plus marks, respectively. The bar indicates the standard deviation within all residues. Atoms without alternative locations were used to draw figures.

As shown in Fig. 4 ▸, a strong resolution dependence of the peak frequency of P atoms was observed. The peak frequency of P atoms dramatically increases to 0.6 at 1.0 Å resolution. The frequency largely differs from that of the simulation with FoFc error and decreases to a similar level in the simulations at 2.5 Å. The average noise using sigmaF was relatively low for all of the resolution ranges studied. We also calculated the peak frequency of C5 of purine or C2 of pyrimidine, which are thought to be relatively rigid compared with the phosphate backbone. Their frequencies are similar to those produced by noise. Thus, the increase in peak frequency in DNA at better resolutions is attributed to the appearance of more minor conformations of the DNA phosphate backbone. We did not see any significant difference in the peak frequencies among A-DNA, B-DNA and Z-DNA. Therefore, the polymorphism of the phosphate backbone does not seem to be attributable to differences in the DNA form. It is notable that even at 1.9 Å resolution, which is close to the median resolution of crystal structures of DNA in the PDB of 1.85 Å as of July 2016, the peak frequency of the P atom is higher than that of the simulation, indicating that some true peaks do exist.

In the case of protein side chains, Lang *et al.* (2010 ▸) reported that secondary peaks at a χ^1^ angle in 2|*F*_o_| − |*F*_c_| maps decrease at lower resolutions. The peak enrichment at rotameric angles, which is an indicator for detecting true minor conformations, diminishes around 2 Å resolution. Therefore, 2 Å seems to be a borderline resolution at which polymorphism may be detected for both proteins and DNA.

### 3.4. Phosphate backbone conformers potentially corresponding to peaks in *m*|*F*_o_| − *D*|*F*_c_| maps   

The phosphate backbones of DNA adopt different conformers such as BI and BII. In this section, we examine the differences in distributions of the peaks arising from the conformers and infer the conformers that correspond to the peaks.

**Figure 5:**
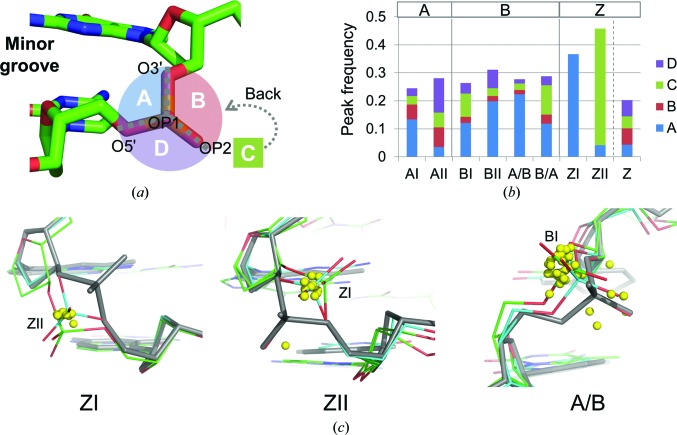
Relationship between the dinucleotide conformer and peak locations. (*a*) Classification of the region around P atoms. The region surrounded by P, O3′, OP1 and O5′ is referred to as subregion A. Similarly, the region surrounded by P, O3′, OP1 and OP2, the region surrounded by P, O3′, OP2 and O5′, and the region surrounded by P, OP1, OP2 and O5′ are referred to as subregions B, C and D, respectively. Subregions A and C are opposed to the minor and major grooves, respectively. (*b*) Peak frequency in each subregion for dinucleotide conformers. ZI and ZII were assigned to R–Y steps for Z-DNA. Z was assigned to Y–R steps. (*c*) Peaks in A/B, ZI and ZII. Yellow spheres indicate peak locations. Each dinucleotide is superimposed on a representative structure of each conformer (grey). The representative structures were PDB entries 460d (A3-A4) for A/B, 3p4j (B10-B11) for ZI and 3p4j (A4-A5) for ZII. (Letters in parentheses indicate chain IDs and residue IDs.) The representative structures were also superimposed on known multiple conformations: green, PDB entry 3wbo (B8-B9), and cyan, PDB entry 4ocb (A8-A9), for ZI; green, PDB entry 3wbo (A4-A5), and cyan, PDB entry 4ocb (A8-A9), for ZII; green, PDB entry 3ggk (A7-A8), and cyan, PDB entry 3ggk (A1-A2), for A/B. C3′, C4′ and O3′ in the residue and P, OP1, OP2, C5′ and O5′ in the next residue were used for superimposition. Only dinucleotides containing DA, DT, DG or DC in both steps were used to draw these figures. If several peaks were found at the location of an atom, the strongest peak was used to draw the figure.

The dinucleotides of our data set were classified according to known dinucleotide conformation clusters: AI, AII, BI, BII, A/B, B/A, ZI (R–Y steps), ZII (R–Y steps) or Z (Y–R steps) (Svozil *et al.*, 2008 ▸). A superimposition of the peaks located around 2.2 Å from P atoms on the representative structure of each conformer is shown in Supplementary Fig. S5. To quantitatively deal with the peak positions, the spatial region around P atoms was divided into four subregions: A, B, C and D (Fig. 5 ▸ *a*). The peak frequencies for these conformers are shown in Fig. 5 ▸(*b*).

The peak frequencies for all conformers of A-DNA and B-DNA were similar to each other. The peaks for ZI and ZII in Z-DNA were more frequently observed than those in A-DNA and B-DNA. In contrast, peaks were less often found for Z in Y–R steps of Z-DNA. Z-DNA is known to be basically composed of repeated structures of a purine and a pyrimidine nucleotide. In another words, Z-DNA consists of repeated structures of more polymorphic phosphate and less polymorphic phosphate.

The distributions of peak locations were largely different among the dinucleotide conformations. For AI, AII, BII and Z the peaks were distributed in all four subregions, indicating that the directions of backbone transitions are not restricted by these conformers. For BI and B/A (B-like for the 5′ nucleotide and A-like for the 3′ nucleotide) the peaks were enriched in subregions A and C and were found less in subregions B and D. Subregions A and C correspond to the sides of the minor and major grooves of phosphates, respectively. Therefore, minor conformations are supposed to be created owing to backbone transitions towards either of the grooves, depending on the structure. In contrast to these conformations, the peaks were localized in subregion A for A/B and ZI, and in subregion C for ZII. All peaks for ZI, in particular, were found only in subregion A. This indicates that the movement of phosphate is strongly restricted to one direction for these three dinucleotide conformations.

The peaks of each of the three conformations were superimposed on known structures with alternative conformations (Fig. 5 ▸ *c*). Polymorphisms between ZI and ZII have been reported for the R–Y step of Z-DNA, as shown in this figure. The locations of the peaks found in ZI were consistent with the locations of P atoms of known alternative ZII conformation. Similarly, the peaks in ZII were found in the location corresponding to P atoms of ZI conformation. Therefore, it is interpreted that the peaks in subregion A for ZI and in subregion C for ZII correspond to transited P atoms. The possibility of transition was confirmed by remodelling a Z-DNA structure solved at ultrahigh resolution (PDB entry 3wbo; Chatake, 2013 ▸). The alternative conformation is highly likely to exist from the electron-density map (Supplementary Fig. S6). The peaks for A/B are not so clearly interpreted compared with those of ZI and ZII. Several structures containing polymorphisms between A/B and BI, which is the most popular B-DNA conformation, have been reported [PDB code (chain + residue ID): 1en3 (A8), 1en8 (A2), 1ene (A2), 3ggi (A2, A8), 3ggk (A2, A8) and 3u89 (A2, A8)]. In all of these structures, P atoms for BI are located in subregion A. Thus, most of the peaks for A/B are interpreted as transitions from A/B to BI.

## 4. Discussion   

### 4.1. Phosphate transitions found in the *m*|*F*_o_| − *D*|*F*_c_| maps   

DNA conformation transitions are important in understanding biological processes. According to current crystallo­graphy, multiple conformations have been assigned to only 6.3% of phosphates in DNA structures solved at resolutions of ≥1.5 Å (calculated from the structures in our data set). The present study showed that there are more conformational variations in crystals than is generally believed. It was found that 27% of phosphates have potential polymorphisms from 1.5 Å resolution electron-density maps. More surprisingly, our analysis demonstrated that 60% of phosphates have potential polymorphisms in 1.0 Å resolution maps.

The densities identified here are weak. In fact, 80% of the peaks observed around 2.2 Å from P atoms were lower than 5σ. When *m*|*F*_o_| − *D*|*F*_c_| maps are calculated after the removal of one of the phosphates from multiple-conformation sites, 80% of peaks corresponding to the removed phosphates appear with a density higher than 15σ (Supplementary Fig. S7). Thus, such peaks with weak density considered in this analysis have not been assigned to any conformation. Even if the individual structures were carefully inspected, most of them could not be modelled in crystal structures using the conventional method. In the present study, the conformations could be assigned to the peaks in A/B, ZI and ZII using prior knowledge of torsion-angle clusters of DNA backbones. Our method may be able to help to build a better model, as exemplified in Supplementary Fig. S6. However, in DNA conformations other than these three conformers, plausible conformations could not be easily assigned to the peaks when a well defined cluster is not observed because the DNA backbone has fewer constraints and can adopt many conformations. In fact, Svozil and coworkers reported a large number of subclusters of di­nucleotide conformations for A-DNA and B-DNA, while they reported only one subcluster for ZI and ZII (Svozil *et al.*, 2008 ▸). In future, these peaks may be interpreted correctly if new classification methods of DNA conformations are developed.

### 4.2. Effects of the local environment on the polymorphism of Z-DNA   

The *m*|*F*_o_| − *D*|*F*_c_| peaks in Z-DNA crystal structures are clearly interpreted as transitions between ZI and ZII. ZI/ZII equilibrium has been also observed in solution by FT-IR (Rauch *et al.*, 2005 ▸) and molecular-dynamics simulations (Ohishi *et al.*, 1997 ▸; Westhof *et al.*, 1986 ▸), after ZI and ZII conformations were observed in the crystal structure of Z-DNA (Wang *et al.*, 1981 ▸). Here, we discuss the effect of DNA modification or local environment of the molecules on ZI/ZII transitions.

As described in §[Sec sec3.4]3.4, the peaks in subregion A for ZI in R–Y steps correspond to the ZII conformation, and those found in subregion C of ZII conformations come from ZI conformations. It was determined that peaks equal to or larger than 3.0σ in subregion A for ZI and subregion C for ZII were considered as potential transition sites of phosphate atoms (listed in Supplementary Table S3). According to this criterion, 57% of R–Y steps analysed have potential transitions or have already been assigned as ZI/ZII transitions.

Firstly, the effects of DNA modifications were examined. Crystal structures with 5-modified pyrimidines such as thymine, 5-bromouracil, 5-bromocytosine and 5-methylcytosine have been reported. As previously mentioned, the ZI conformation is preferred for 5-bromocytosine (Chevrier *et al.*, 1986 ▸). For all pyrimidine 5-modifications, peaks corresponding to potential ZI/ZII transitions were observed as in the unmodified cytidine. Therefore, 5-modifications of pyrimidine do not seem to affect the ZI/ZII transitions of Z-DNA in general. The observation is consistent with that obtained from a previous MD study, in which 5-methylation of Z-DNA did not affect the ZI/ZII transition (Temiz *et al.*, 2012 ▸). 5-Methylation is known to stabilize B–Z transitions (Rich, 1984 ▸). Previous crystallographic studies showed that 5-methylation did not affect the global structures of DNA, with the exception of subtle changes in helical twist angles (Fujii *et al.*, 1982 ▸; Ho & Mooers, 1997 ▸). It was suggested that 5-methylation is associated with a relative destabilization of the B-DNA structure and stabilization through hydrophobic bonding of Z-DNA structures. Therefore, an equilibrium between ZI and ZII of phosphates would not be directly related to B–Z transitions.

**Figure 6:**
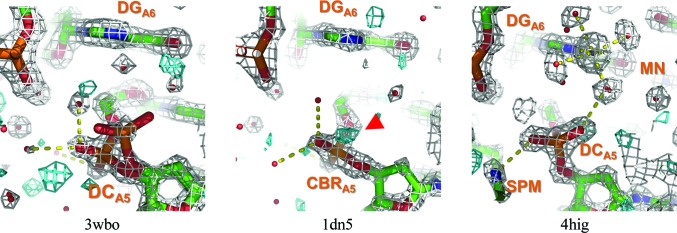
Electron-density maps in cation/polyamine-binding sites. The 2*m*|*F*_o_| − *D*|*F*_c_| maps are shown in grey and the *m*|*F*_o_| − *D*|*F*_c_| maps are shown in cyan. The contour levels of 2*m*|*F*_o_| − *D*|*F*_c_| and *m*|*F*_o_| − *D*|*F*_c_| maps are 1σ and 2.75σ, respectively. Peaks in the *m*|*F_o_*| − *D*|*F*_c_| maps potentially corresponding to ZII are highlighted by red arrows. Yellow dotted lines indicate hydrogen bonds or coordination bonds. Residue names are shown in orange, followed by the chain ID and residue ID as subscripts. The resolution used to draw these maps was 1.5 Å. Other maps are shown in Supplementary Fig. S8.

A number of Z-DNA crystal structures of d(CGCGCG) have been solved in the same crystal system: *P*2_1_2_1_2_1_ with unit-cell parameters *a* = 17.8, *b* = 31.2, *c* = 44.3 Å. Gessner *et al.* (1989 ▸) first reported that guanine N7 at the sixth residue in chain A is a metal cation coordination site in the crystal structure. Through closer observation, it was found that cations are intimately associated with polymorphism of the fifth residue in the same chain. The metal coordination–polyamine interactions of the fifth residue are summarized in Table 1 ▸. Electron-density maps for the representative structures of the fifth phosphates are shown in Fig. 6 ▸ and those for the remaining structures in Supplementary Fig. S8. As shown in Table 1 ▸, when guanine N7 at the sixth residue is directly coordinated by cations, the ZI/ZII polymorphism at the fifth residue tends to be suppressed so that the ZII conformation is preferable, with the exception of PDB entry 1vro, which has phosphoroselenoate at the second residue of chain A. In these structures, five water molecules are octahedrally coordinated to the metal ion. One of the water molecules stabilizes the ZII conformation of the fifth phosphate by interaction with the OP1 atom of the phosphate, as shown for PDB entry 4hig in Fig. 6 ▸. This hydrogen-bond network disappears when no cation is located at this site (PDB entry 1dn5 in Fig. 6 ▸). Interactions between polyamines and the fifth phosphates were sometimes observed in addition to metal binding. These interactions cooperatively stabilize ZII conformations of the phosphates. However, PDB entry 3p4j apparently does not have any metal ions at this site, but ZI/ZII polymorphism of the fifth residue is suppressed by water molecules. In this structure, ‘octahedral’ water molecules are found at this site. A certain contaminant ion might exist at this site, because a hydrogen-bonding structure by water is ideally ‘tetrahedral’. PDB entry 3wbo (Fig. 6 ▸) is an interesting structure. In this structure, ZI/ZII transitions were assigned by the authors (the occupancy for ZI is 0.67 and that for ZII is 0.33), indicating that the transitions in this structure are more obvious than those in other structures. This specimen was thoroughly de­mineralized using liquid chromatography and no divalent cations were included in the crystallization condition (Chatake, 2013 ▸). The removal of divalent cations induces ZI/ZII transitions. The previous CD spectroscopic study showed that divalent cations such as Ca^2+^ and Mg^2+^ stabilize Z′ conformations of poly(GC) in solution (Harder & Johnson, 1990 ▸). They suggested that the Z′ form in CD is related to the ZII form in crystals. Metal coordination to guanine N7 may stabilize ZII conformations.

It has been pointed out that conventional refinement methods do not always provide us with an accurate view of crystallographic heterogeneity, with possible minor conformations being overlooked (DePristo *et al.*, 2004 ▸; Lang *et al.*, 2010 ▸). The present study showed that a large amount of structural information for phosphate transitions is found in the DNA crystal structures by introducing a new analytic procedure. This does not result from placing the focus only on ultrahigh-resolution structures. In fact, the resolution used in the present study, 1.5 Å, represents that of 20% of DNA structures in the PDB.

High-intensity X-ray sources such as synchrotrons and XFELs have been developed and have recently become available. Such resources produce more high-resolution data, indicating that the electron densities of minor conformations may be made more visible. To extract the full information from such data, further novel refinement methods will be desirable.

## 5. Conclusion   

DNA conformations are important for molecular recognition of DNA-binding proteins. However, multiple conformations have been assigned to only 6.3% of the phosphates in crystal structures solved at a resolution equal to or higher than 1.5 Å. In the present study, a comprehensive analysis demonstrated that conformational variations of DNA are more extensive than recorded in the coordinates of crystal data in the PDB. Based on the unassigned densities of DNA crystal structures obtained by recalculating the *m*|*F*_o_| − *D*|*F*_c_| maps, peaks were found in the vicinity of 27% of phosphates. It was also found that the frequency of peak appearance is strongly dependent on the X-ray resolution. Interestingly, more than half of P atoms accompany the *m*|*F*_o_| − *D*|*F*_c_| peaks in structures at 1.0 Å resolution. Since the peaks for A/B, ZI and ZII conformers are clearly localized, conformations could be assigned to them. In addition, a relationship between the local environment and transitions of phosphate backbone in Z-DNA structures was found.

Our analysis indicates that the electron-density maps contain a large amount of structural information and that phosphate backbones assume quite polymorphic conformations even in crystals. Such information provides us with a basis to deepen our understanding of the nature of DNA conformations.

## Supplementary Material

Supplementary Figures and Tables.. DOI: 10.1107/S2059798317007707/kw5135sup1.pdf

## Figures and Tables

**Table 1:** ZI/ZII transitions found in the cation/polyamine-binding sites in *P*2_1_2_1_2_1_ form Z-DNA

			ZI/ZII transition			
PDB code	Chain + residue ID	Residue name	Assigned in PDB	From peak detection	Peak intensity for ZI/ZII†	Metal/polyamine binding
1d39	A5	DC	ZII	ZII		Cu(H_2_O)_5_
2elg	A5	DC	ZII	ZII		Mg(H_2_O)_5_
1ick	A5	DC	ZII	ZII		Mg(H_2_O)_5_/SPM
1vro	B111	DC	ZII	ZI/ZII	3.85	Mg(H_2_O)_5_/SPM
4hif	A5	DC	ZII	ZII		Zn(H_2_O)_5_
4hig	A5	DC	ZII	ZII		Mn(H_2_O)_5_/SPK
3p4j	A5	DC	ZII	ZII		Octahedral water structure [potentially metal-(H_2_O)_5_ binding]/SPM
1d41	A5	5CM	ZII	ZI/ZII	3.07	
1d76	A5	DC	ZII	ZI/ZII	3.47	
1dj6	A5	DC	ZII	ZI/ZII	5.20	
1dn5	A5	CBR	ZI	ZI/ZII	3.60	
2obz	A5	BRU	ZII	ZI/ZII	4.40	
3wbo	A5	DC	ZI/ZII	ZI/ZII	—	

† Peaks were extracted from the *m*|*F*_o_| − *D*|*F*_c_| maps at 1.5 Å resolution. Peak intensity for ZI/ZII indicates the intensity of a peak found in subregion A for ZI and subregion C for ZII, respectively. Every peak listed here is the highest peak at a distance within 2.2 Å of the P atom.

## References

[bb1] Adams, P. D. *et al.* (2010). *Acta Cryst.* D**66**, 213–221.

[bb44] Brzezinski, K., Brzuszkiewicz, A., Dauter, M., Kubicki, M., Jaskolski, M. & Dauter, Z. (2011). *Nucleic Acids Res.* **39**, 6238–6248. 10.1093/nar/gkr202 PMC3152349 21459852

[bb2] Burnley, B. T., Afonine, P. V., Adams, P. D. & Gros, P. (2012). *Elife*, **1**, e00311. 10.7554/eLife.00311 PMC3524795 23251785

[bb3] Chatake, T. (2013). *J. Synchrotron Rad.* **20**, 864–868. 10.1107/S0909049513020773 PMC3795545 24121329

[bb4] Chevrier, B., Dock, A. C., Hartmann, B., Leng, M., Moras, D., Thuong, M. T. & Westhof, E. (1986). *J. Mol. Biol.* **188**, 707–719. 10.1016/s0022-2836(86)80016-x 3735433

[bb5] DePristo, M. A., de Bakker, P. I. & Blundell, T. L. (2004). *Structure*, **12**, 831–838. 10.1016/j.str.2004.02.031 15130475

[bb6] Djuranovic, D. & Hartmann, B. (2005). *Biophys. J.* **89**, 2542–2551. 10.1529/biophysj.104.057109 PMC1366753 16055534

[bb7] Egli, M., Tereshko, V., Teplova, M., Minasov, G., Joachimiak, A., Sanishvili, R., Weeks, C. M., Miller, R., Maier, M. A., An, H., Cook, P. D. & Manoharan, M. (1998). *Biopolymers*, **48**, 234–252. 10.1002/(SICI)1097-0282(1998)48:4<234::AID-BIP4>3.0.CO;2-H 10699842

[bb8] Fraser, J. S., van den Bedem, H., Samelson, A. J., Lang, P. T., Holton, J. M., Echols, N. & Alber, T. (2011). *Proc. Natl Acad. Sci. USA*, **108**, 16247–16252. 10.1073/pnas.1111325108 PMC3182744 21918110

[bb9] Fujii, S., Wang, A. H.-J., van der Marel, G., van Boom, J. H. & Rich, A. (1982). *Nucleic Acids Res.* **10**, 7879–7892. 10.1093/nar/10.23.7879 PMC327053 7155900

[bb10] Gessner, R. V., Frederick, C. A., Quigley, G. J., Rich, A. & Wang, A. H.-J. (1989). *J. Biol. Chem.* **264**, 7921–7935. 10.2210/pdb1dcg/pdb 2722771

[bb11] Harder, M. E. & Johnson, W. C. Jr (1990). *Nucleic Acids Res.* **18**, 2141–2148. 10.1093/nar/18.8.2141 PMC330694 2336392

[bb12] Henderson, R. & Moffat, J. K. (1971). *Acta Cryst.* B**27**, 1414–1420.

[bb13] Ho, P. S. & Mooers, B. H. (1997). *Biopolymers*, **44**, 65–90. 10.1002/(SICI)1097-0282(1997)44:1<65::AID-BIP5>3.0.CO;2-Y 19174848

[bb14] Kielkopf, C. L., Ding, S., Kuhn, P. & Rees, D. C. (2000). *J. Mol. Biol.* **296**, 787–801. 10.1006/jmbi.1999.3478 10677281

[bb15] Knight, J. L., Zhou, Z., Gallicchio, E., Himmel, D. M., Friesner, R. A., Arnold, E. & Levy, R. M. (2008). *Acta Cryst.* D**64**, 383–396. 10.1107/S090744490800070X PMC2631124 18391405

[bb16] Lang, P. T., Holton, J. M., Fraser, J. S. & Alber, T. (2014). *Proc. Natl Acad. Sci. USA*, **111**, 237–242.

[bb17] Lang, P. T., Ng, H. L., Fraser, J. S., Corn, J. E., Echols, N., Sales, M., Holton, J. M. & Alber, T. (2010). *Protein Sci.* **19**, 1420–1431. 10.1002/pro.423 PMC2974833 20499387

[bb18] Levin, E. J., Kondrashov, D. A., Wesenberg, G. E. & Phillips, G. N. Jr (2007). *Structure*, **15**, 1040–1052. 10.1016/j.str.2007.06.019 PMC2039884 17850744

[bb19] Lu, X.-J. & Olson, W. K. (2003). *Nucleic Acids Res.* **31**, 5108–5121. 10.1093/nar/gkg680 PMC212791 12930962

[bb21] Ohishi, H., Nakanishi, I. & Tomita, K. (1997). *Biochem. Biophys. Res. Commun.* **236**, 146–150. 10.1006/bbrc.1997.6917 9223442

[bb22] Olson, W. K., Gorin, A. A., Lu, X.-J., Hock, L. M. & Zhurkin, V. B. (1998). *Proc. Natl Acad. Sci. USA*, **95**, 11163–11168. 10.1073/pnas.95.19.11163 PMC21613 9736707

[bb23] Painter, J. & Merritt, E. A. (2004). *J. Appl. Cryst.* **37**, 174–178.

[bb24] Rauch, C., Pichler, A., Trieb, M., Wellenzohn, B., Liedl, K. R. & Mayer, E. (2005). *J. Biomol. Struct. Dyn.* **22**, 595–614. 10.1080/07391102.2005.10507029 15702931

[bb25] Read, R. J. (1986). *Acta Cryst.* A**42**, 140–149.

[bb26] Rich, A. (1984). *DNA Methylation*, edited by A. Razin, H. Cedar & A. D. Riggs, pp. 279–292. New York: Springer-Verlag.

[bb27] Robertson, J. C. & Cheatham, T. E. III (2015). *J. Phys. Chem. B*, **119**, 14111–14119. 10.1021/acs.jpcb.5b08486 26482568

[bb28] Rohs, R., West, S. M., Sosinsky, A., Liu, P., Mann, R. S. & Honig, B. (2009). *Nature (London)*, **461**, 1248–1253. 10.1038/nature08473 PMC2793086 19865164

[bb29] Sarai, A. & Kono, H. (2005). *Annu. Rev. Biophys. Biomol. Struct.* **34**, 379–398. 10.1146/annurev.biophys.34.040204.144537 15869395

[bb30] Schneider, B., Gelly, J.-C., de Brevern, A. G. & Černý, J. (2014). *Acta Cryst.* D**70**, 2413–2419. 10.1107/S1399004714014631 PMC4157449 25195754

[bb31] Schneider, B., Neidle, S. & Berman, H. M. (1997). *Biopolymers*, **42**, 113–124. 10.1002/(sici)1097-0282(199707)42:1<113::aid-bip10>3.0.co;2-o 19350745

[bb33] Svozil, D., Kalina, J., Omelka, M. & Schneider, B. (2008). *Nucleic Acids Res.* **36**, 3690–3706. 10.1093/nar/gkn260 PMC2441783 18477633

[bb34] Temiz, N. A., Donohue, D. E., Bacolla, A., Luke, B. T. & Collins, J. R. (2012). *PLoS One*, **7**, e35558. 10.1371/journal.pone.0035558 PMC3328458 22530050

[bb35] Terwilliger, T. C., Grosse-Kunstleve, R. W., Afonine, P. V., Adams, P. D., Moriarty, N. W., Zwart, P., Read, R. J., Turk, D. & Hung, L.-W. (2007). *Acta Cryst.* D**63**, 597–610. 10.1107/S0907444907009791 PMC2483474 17452785

[bb36] Tisné, C., Hantz, E., Hartmann, B. & Delepierre, M. (1998). *J. Mol. Biol.* **279**, 127–142. 10.1006/jmbi.1998.1757 9636705

[bb37] Tisné, C., Hartmann, B. & Delepierre, M. (1999). *Biochemistry*, **38**, 3883–3894. 10.1021/bi982402d 10194299

[bb38] Vitkup, D., Ringe, D., Karplus, M. & Petsko, G. A. (2002). *Proteins*, **46**, 345–354. 10.1002/prot.10035 11835510

[bb39] Wakamori, M., Fujii, Y., Suka, N., Shirouzu, M., Sakamoto, K., Umehara, T. & Yokoyama, S. (2015). *Sci. Rep.* **5**, 17204. 10.1038/srep17204 PMC4660432 26607036

[bb40] Wang, A. J., Quigley, G. J., Kolpak, F. J., van der Marel, G., van Boom, J. H. & Rich, A. (1981). *Science*, **211**, 171–176. 10.1126/science.7444458 7444458

[bb41] Wecker, K., Bonnet, M. C., Meurs, E. F. & Delepierre, M. (2002). *Nucleic Acids Res.* **30**, 4452–4459. 10.1093/nar/gkf559 PMC137123 12384592

[bb42] Westhof, E., Chevrier, B., Gallion, S. L., Weiner, P. K. & Levy, R. M. (1986). *J. Mol. Biol.* **191**, 699–712. 10.1016/0022-2836(86)90454-7 3806679

[bb43] Winn, M. D. *et al.* (2011). *Acta Cryst.* D**67**, 235–242.

