# Supplementary material for: DNA conformational transitions inferred from re-evaluation of m|F o| − D|F c| electron-density maps
Source: Acta Crystallogr D Struct Biol. 2017 Jun 22;73(Pt 7):600–8. doi: 10.1107/S2059798317007707 (PMC5505156; doi:10.1107/S2059798317007707)
Supplement: Supplementary file 1 [file d-73-00600-sup1.pdf]

STRUCTURAL BIOLOGY

Volume 73 (2017)

Supporting information for article:

DNA conformational transitions inferred from re-evaluation of $m|F_o| - D|F_c|$ electron-density maps

Tomoko Sunami, Toshiyuki Chatake and Hidetoshi Kono

Table S1 e/A^3 vs σ in m|Fo|-D|Fc| maps at 1.5Å resolution

PDB ID	e/A^3/σ	NDB classification
4LTF	0.07138	Other duplex
4KW0	0.0719	B-DNA
4MKW	0.07912	B-DNA
4F3U	0.08166	B-DNA
4C63	0.09202	B-DNA
4C64	0.09277	B-DNA
1XUX	0.09473	A-DNA
1D79	0.09484	A-DNA
2FII	0.0954	B-DNA
2O1I	0.09551	B-DNA
4I9V	0.09662	B-DNA
1XUW	0.09987	A-DNA
3V07	0.10008	A-DNA
1FQ2	0.1012	B-DNA
1IH6	0.10233	A-DNA
1R3G	0.10339	A-DNA
4C5X	0.10347	B-DNA
4FS6	0.10428	Z-DNA
3QRN	0.10612	B-DNA
1N1O	0.10825	B-DNA
4E8S	0.11157	B-DNA
2NSK	0.11159	A-DNA
1MLX	0.11281	A-DNA
3MBS	0.11422	-
4AGZ	0.11575	B-DNA
1XCS	0.11703	B-DNA
3U89	0.11755	B-DNA
3OPI	0.11773	B-DNA
4GJU	0.11885	B-DNA
463D	0.11928	B-DNA
460D	0.12014	B-DNA
1PJO	0.12132	-
4AH0	0.12295	B-DNA
2FIH	0.123	B-DNA
1D78	0.12343	A-DNA
3UYB	0.12368	B-DNA
1EI4	0.1237	B-DNA

4I1G	0.12459	A-DNA
4LTI	0.1255	Other duplex
1I0G	0.12723	A-DNA
3C1P	0.1277	-
1M69	0.12771	B-DNA
2DLJ	0.12779	A-DNA
476D	0.12954	B-DNA
4LTK	0.13006	Other duplex
403D	0.13033	B-DNA
2HC7	0.13036	A-DNA
2FIJ	0.13177	A-DNA
3OMJ	0.13218	B-DNA
1R68	0.13275	B-DNA
224D	0.13357	Other duplex
3U08	0.13471	B-DNA
3EY3	0.13498	B-DNA
1PJG	0.1351	-
1Y8V	0.1357	A-DNA
2B3E	0.13592	B-DNA
4H5A	0.13604	B-DNA
1FD5	0.13696	B-DNA
1DPN	0.13753	B-DNA
3LTR	0.13848	A-DNA
4FP6	0.13857	-
1Y8L	0.13991	A-DNA
1ICK	0.13992	Z-DNA
3P4J	0.14001	Z-DNA
366D	0.1423	Other duplex
191D	0.14267	Quadruplex
4F4N	0.1429	A-DNA
2DPB	0.14303	B-DNA
3U2N	0.1435	B-DNA
436D	0.14373	B-DNA
1O0K	0.14419	Quadruplex
1D9R	0.14427	B-DNA
3IJK	0.14466	A-DNA
1DC0	0.14577	Other duplex
1I0N	0.14598	A-DNA
3U0U	0.14816	B-DNA
3U05	0.14859	B-DNA

4HIF	0.14973	Z-DNA
3NYP	0.15091	Quadruplex
145D	0.15136	Z-DNA
440D	0.15228	A-DNA
1KGK	0.15272	A-DNA
3LTU	0.15273	A-DNA
3EY2	0.15313	A-DNA
1Z3F	0.15405	B-DNA
4L26	0.15413	Other duplex
355D	0.1543	B-DNA
3IKI	0.15493	A-DNA
3NZ7	0.1554	Quadruplex
1Z7I	0.15594	A-DNA
427D	0.15602	Other duplex
1QYL	0.15702	Quadruplex
1M77	0.15766	A-DNA
3IFI	0.15779	A-DNA
3HG8	0.15802	A-DNA
1JES	0.15929	Z-DNA
3FT6	0.15946	B-DNA
455D	0.16086	B-DNA
1G4Q	0.16135	-
3GGK	0.16267	B-DNA
2PLO	0.16275	A-DNA
431D	0.16458	B-DNA
1PUY	0.16608	Other duplex
3GGI	0.16724	B-DNA
1MF5	0.16752	Quadruplex
4OCB	0.16759	Z-DNA
3I5E	0.16816	B-DNA
1VRO	0.16853	Z-DNA
1D76	0.17212	Z-DNA
1DPL	0.17245	A-DNA
4L25	0.17252	Other duplex
3TVB	0.17253	Quadruplex
1I0P	0.1733	A-DNA
3FL6	0.17676	B-DNA
1WOE	0.17991	Z-DNA
1D8G	0.18263	B-DNA
1EN9	0.18314	B-DNA

1O55	0.18393	Single stranded
1D8X	0.18606	B-DNA
1NVY	0.18618	B-DNA
3WBO	0.1863	Z-DNA
1ENN	0.19115	B-DNA
1EN3	0.19147	B-DNA
1EN8	0.19428	B-DNA
2O4F	0.19525	Quadruplex
362D	0.19719	Z-DNA
2F8W	0.20114	Z-DNA
4HIG	0.20205	Z-DNA
1OMK	0.20597	Z-DNA
1ENE	0.20805	B-DNA
3I5L	0.20834	B-DNA
4FS5	0.21138	Z-DNA
1DN5	0.21182	Z-DNA
1EM0	0.21829	-
1D39	0.22028	Z-DNA
1D41	0.22214	Z-DNA
2OBZ	0.22647	Z-DNA
1DJ6	0.23616	Z-DNA
1I0K	0.25249	A-DNA
1ZF1	0.25949	A-DNA
1ZF8	0.26877	A-DNA
1ZF9	0.28095	A-DNA
1I0J	0.28734	A-DNA
1ZF0	0.29727	B-DNA
1I0M	0.30117	A-DNA
1ZF7	0.31096	B-DNA
2ELG	0.31174	Z-DNA
1ZF6	0.32999	A-DNA
284D	0.33783	Quadruplex

Table S2 PDB ID, resolution, R_{work}, R_{free} and NDB classification

PDB ID	Resolution(Å)	R_{work}	R_{free}	NDB Classification
102D	1.31	0.248	0.252	B-DNA
109D	1.80	0.219	0.238	B-DNA
110D	1.25	0.209	0.211	Other duplex
116D	2.25	0.230	0.266	A-DNA
118D	1.50	0.179	0.196	A-DNA
119D	1.80	0.205	0.198	B-DNA
126D	1.50	0.151	0.146	B-DNA
133D	1.53	0.202	0.205	Z-DNA
145D	1.42	0.201	0.223	Z-DNA
150D	2.40	0.220	0.246	B-DNA
158D	1.67	0.222	0.232	B-DNA
166D	2.15	0.147	0.155	B-DNA
178D	1.92	0.219	0.262	B-DNA
182D	2.20	0.213	0.212	Other duplex
184D	1.25	0.157	0.185	Quadruplex
191D	1.93	0.175	0.193	Quadruplex
196D	1.50	0.239	0.253	B-DNA
1A2E	1.10	0.182	0.192	Other duplex
1BD1	2.10	0.227	0.225	B-DNA
1CGC	1.10	0.232	0.234	B-DNA
1D23	2.50	0.214	0.240	B-DNA
1D24	1.68	0.201	0.235	Z-DNA
1D26	1.80	0.177	0.196	A-DNA
1D29	1.85	0.231	0.252	B-DNA
1D30	1.50	0.205	0.183	B-DNA
1D39	2.00	0.232	0.244	Z-DNA
1D41	2.00	0.198	0.230	Z-DNA

1D43	2.45	0.178	0.236	B-DNA
1D56	1.60	0.225	0.231	B-DNA
1D60	1.90	0.247	0.293	B-DNA
1D63	1.90	0.219	0.208	B-DNA
1D64	1.57	0.202	0.215	B-DNA
1D65	1.60	0.192	0.202	B-DNA
1D76	2.45	0.182	0.192	Z-DNA
1D78	2.00	0.175	0.199	A-DNA
1D79	2.00	0.207	0.237	A-DNA
1D80	1.95	0.218	0.212	B-DNA
1D89	1.02	0.153	0.180	B-DNA
1D8G	1.60	0.195	0.212	B-DNA
1D8X	1.90	0.211	0.222	B-DNA
1D9R	1.67	0.187	0.226	B-DNA
1DA3	1.80	0.229	0.248	B-DNA
1DA9	1.30	0.142	0.160	Other duplex
1DC0	1.18	0.108	0.119	Other duplex
1DCR	2.16	0.223	0.249	B-DNA
1DCV	1.92	0.168	0.213	B-DNA
1DCW	2.00	0.167	0.165	Other duplex
1DJ6	1.50	0.171	0.193	Z-DNA
1DL8	1.00	0.219	0.223	Other duplex
1DN4	2.00	0.172	0.201	Z-DNA
1DN5	1.67	0.187	0.208	Z-DNA
1DNH	2.25	0.166	0.161	B-DNA
1DNZ	1.60	0.231	0.273	A-DNA
1DPL	1.30	0.205	0.219	A-DNA
1DPN	1.62	0.165	0.159	B-DNA
1DVL	1.80	0.239	0.257	B-DNA

1EFO	1.49	0.184	0.231	
1EHV	2.10	0.164	0.195	B-DNA
1EI4	1.27	0.206	0.217	B-DNA
1EM0	0.95	0.096	0.099	
1EN3	1.53	0.201	0.184	B-DNA
1EN8	1.60	0.175	0.213	B-DNA
1EN9	2.30	0.232	0.271	B-DNA
1ENE	1.75	0.215	0.248	B-DNA
1ENN	1.60	0.214	0.231	B-DNA
1F6E	1.27	0.207	0.227	A-DNA
1F6I	1.60	0.175	0.194	Other duplex
1F6J	1.95	0.224	0.264	Other duplex
1FD5	1.60	0.205	0.205	B-DNA
1FDG	1.65	0.172	0.170	B-DNA
1FHY	2.50	0.155	0.196	B-DNA
1FHZ	2.21	0.173	0.200	B-DNA
1FIX	1.70	0.261	0.264	
1FN1	2.20	0.160	0.171	B-DNA
1FN2	2.35	0.144	0.161	B-DNA
1FQ2	2.50	0.187	0.207	B-DNA
1FTD	2.38	0.195	0.236	B-DNA
1G00	0.98	0.148	0.143	A-DNA
1G4Q	2.40	0.149	0.178	
1GQU	1.58	0.194	0.221	Other duplex
1HQ7	1.41	0.169	0.178	B-DNA
1HZS	1.15	0.174	0.201	Other duplex
1I0F	2.40	0.221	0.199	A-DNA
1I0G	1.60	0.219	0.231	A-DNA
1I0J	2.28	0.177	0.208	A-DNA

1I0K	1.10	0.165	0.186	A-DNA
1I0M	1.85	0.180	0.159	A-DNA
1I0N	1.13	0.122	0.146	A-DNA
1I0O	0.98	0.126	0.141	A-DNA
1I0P	2.20	0.204	0.216	A-DNA
1I1P	1.50	0.248	0.228	B-DNA
1I3T	1.55	0.184	0.178	B-DNA
1I47	1.20	0.161	0.179	B-DNA
1ICK	2.05	0.233	0.261	Z-DNA
1IH1	2.21	0.231	0.265	B-DNA
1IH3	1.25	0.203	0.205	A-DNA
1IH4	1.20	0.232	0.239	A-DNA
1IH6	1.10	0.244	0.254	A-DNA
1IHH	1.50	0.135	0.109	B-DNA
1IKK	1.61	0.200	0.229	B-DNA
1J8L	2.20	0.205	0.262	B-DNA
1JB8	1.94	0.253	0.298	
1JES	2.03	0.156	0.210	Z-DNA
1JPQ	1.20	0.223	0.216	Quadruplex
1JRN	1.50	0.164	0.171	Quadruplex
1JTL	1.10	0.164	0.181	B-DNA
1JUC	1.63	0.210	0.224	Other duplex
1JUX	0.75	0.085	0.086	B-DNA
1K2L	2.20	0.264	0.272	Other duplex
1K2Z	1.56	0.184	0.183	B-DNA
1K8P	1.60	0.168	0.176	Quadruplex
1KCI	1.50	0.168	0.186	B-DNA
1KF1	2.35	0.233	0.215	Quadruplex
1KGK	1.99	0.202	0.215	A-DNA

1L1H	2.35	0.223	0.269	Quadruplex
1L4J	2.01	0.249	0.260	Other duplex
1L6B	2.15	0.173	0.214	Other duplex
1LEX	1.90	0.228	0.265	B-DNA
1LJX	1.90	0.187	0.218	Z-DNA
1M69	2.21	0.162	0.183	B-DNA
1M6F	2.40	0.209	0.216	B-DNA
1M6G	1.60	0.171	0.195	Other duplex
1M6R	1.40	0.180	0.203	Z-DNA
1M77	0.74	0.142	0.148	A-DNA
1MF5	1.80	0.191	0.193	Quadruplex
1MLX	2.30	0.255	0.264	A-DNA
1N1O	2.50	0.149	0.204	B-DNA
1N5C	1.45	0.219	0.246	B-DNA
1NAB	2.13	0.177	0.178	B-DNA
1NGT	1.65	0.179	0.212	B-DNA
1NQS	1.60	0.160	0.161	B-DNA
1NR8	1.81	0.199	0.216	Other duplex
1NT8	2.40	0.224	0.213	B-DNA
1NVN	2.40	0.185	0.185	B-DNA
1NVY	2.10	0.225	0.234	B-DNA
1NZG	2.15	0.218	0.214	A-DNA
1O0K	1.60	0.209	0.216	Quadruplex
1O55	2.40	0.205	0.260	Single stranded
1OMK	2.04	0.210	0.234	Z-DNA
1P1Y	1.54	0.201	0.218	Other duplex
1P4Y	2.50	0.182	0.237	B-DNA
1P4Z	2.50	0.233	0.216	B-DNA
1P54	2.00	0.240	0.275	B-DNA

1PJG	1.53	0.181	0.220	
1PJO	1.30	0.213	0.200	
1PRP	2.25	0.173	0.171	B-DNA
1PUY	1.30	0.160	0.190	Other duplex
1QC1	1.51	0.202	0.211	B-DNA
1QDA	1.20	0.183	0.186	Other duplex
1QV4	2.50	0.226	0.191	B-DNA
1QV8	2.00	0.261	0.273	B-DNA
1QYL	2.00	0.229	0.232	Quadruplex
1R2O	1.60	0.170	0.203	Quadruplex
1R3G	1.18	0.166	0.191	A-DNA
1R3Z	2.22	0.191	0.232	A-DNA
1R41	2.30	0.197	0.222	Other duplex
1R68	1.82	0.210	0.207	B-DNA
1RQY	1.50	0.179	0.181	B-DNA
1S1K	1.70	0.171	0.170	B-DNA
1S1L	1.77	0.224	0.231	Other duplex
1S23	0.98	0.156	0.162	B-DNA
1S2R	1.20	0.157	0.182	B-DNA
1S45	1.30	0.171	0.176	Quadruplex
1S47	2.50	0.247	0.299	Quadruplex
1UB8	1.75	0.230	0.227	Other duplex
1UE3	1.55	0.211	0.229	Other duplex
1UE4	1.24	0.161	0.183	Other duplex
1VAQ	1.60	0.217	0.234	A-DNA
1VRO	1.85	0.197	0.182	Z-DNA
1VZK	1.05	0.264	0.258	B-DNA
1WOE	0.95	0.110	0.115	Z-DNA
1WQY	1.50	0.168	0.191	B-DNA

1WV5	1.80	0.196	0.166	A-DNA
1XA2	2.50	0.149	0.172	Z-DNA
1XCS	1.38	0.241	0.247	B-DNA
1XCU	1.80	0.199	0.244	B-DNA
1XJX	1.55	0.188	0.204	A-DNA
1XJY	1.40	0.175	0.191	A-DNA
1XUW	2.10	0.227	0.252	A-DNA
1XUX	1.90	0.185	0.215	A-DNA
1Y7F	1.63	0.153	0.171	A-DNA
1Y84	1.80	0.249	0.267	A-DNA
1Y86	2.39	0.163	0.168	A-DNA
1Y8L	1.94	0.181	0.221	A-DNA
1Y8V	1.24	0.106	0.122	A-DNA
1Y9F	1.25	0.156	0.194	A-DNA
1Y9S	1.60	0.215	0.213	A-DNA
1YB9	1.65	0.210	0.220	A-DNA
1YBC	1.20	0.161	0.168	A-DNA
1Z3F	2.50	0.223	0.210	B-DNA
1Z5T	2.50	0.230	0.270	B-DNA
1Z7I	1.85	0.205	0.238	A-DNA
1Z8V	1.60	0.180	0.166	B-DNA
1ZEW	1.90	0.165	0.183	B-DNA
1ZEX	1.41	0.138	0.135	A-DNA
1ZEY	1.60	0.162	0.184	A-DNA
1ZEZ	1.55	0.221	0.244	B-DNA
1ZF0	1.60	0.180	0.191	B-DNA
1ZF1	1.78	0.199	0.216	A-DNA
1ZF2	1.30	0.201	0.226	B-DNA
1ZF3	1.65	0.237	0.238	B-DNA

1ZF4	1.61	0.201	0.221	B-DNA
1ZF6	1.70	0.227	0.240	A-DNA
1ZF7	2.20	0.183	0.229	B-DNA
1ZF8	1.80	0.230	0.274	A-DNA
1ZF9	2.20	0.161	0.165	A-DNA
1ZFA	1.88	0.217	0.267	A-DNA
1ZFB	2.20	0.213	0.285	B-DNA
1ZFC	1.32	0.143	0.183	B-DNA
1ZFM	1.28	0.185	0.219	B-DNA
1ZPH	1.25	0.157	0.160	B-DNA
1ZPI	1.65	0.162	0.183	B-DNA
211D	2.00	0.176	0.198	Z-DNA
218D	1.65	0.186	0.217	B-DNA
223D	2.20	0.222	0.261	Z-DNA
224D	1.54	0.157	0.162	Other duplex
227D	1.35	0.230	0.229	B-DNA
233D	2.13	0.165	0.188	B-DNA
242D	1.60	0.238	0.241	Z-DNA
249D	1.90	0.222	0.227	B-DNA
251D	2.26	0.220	0.248	B-DNA
254D	1.65	0.197	0.210	A-DNA
256D	2.00	0.245	0.279	A-DNA
257D	1.57	0.214	0.227	A-DNA
258D	1.36	0.207	0.199	B-DNA
263D	2.10	0.192	0.241	B-DNA
265D	1.60	0.210	0.205	B-DNA
266D	1.65	0.247	0.252	B-DNA
267D	1.94	0.200	0.233	B-DNA
268D	1.17	0.156	0.176	B-DNA

269D	1.92	0.248	0.285	B-DNA
270D	2.10	0.225	0.261	B-DNA
271D	1.54	0.218	0.220	B-DNA
272D	1.00	0.193	0.191	B-DNA
275D	0.83	0.140	0.148	A-DNA
284D	1.65	0.231	0.253	Quadruplex
285D	1.80	0.205	0.217	B-DNA
286D	1.30	0.192	0.232	B-DNA
287D	2.00	0.204	0.239	B-DNA
289D	1.30	0.177	0.192	B-DNA
297D	1.10	0.111	0.127	B-DNA
298D	2.50	0.180	0.195	B-DNA
2A7E	1.61	0.236	0.266	A-DNA
2AVH	1.00	0.123	0.133	Quadruplex
2AVJ	2.23	0.210	0.203	Quadruplex
2AXB	2.20	0.198	0.243	A-DNA
2B0K	1.86	0.222	0.217	B-DNA
2B1C	2.20	0.226	0.229	A-DNA
2B1D	1.40	0.177	0.198	B-DNA
2B2B	2.10	0.173	0.183	B-DNA
2B3E	2.00	0.217	0.243	B-DNA
2D25	2.50	0.210	0.253	B-DNA
2DES	1.63	0.168	0.168	Other duplex
2DLJ	1.60	0.225	0.239	A-DNA
2DP7	1.80	0.169	0.208	B-DNA
2DPB	1.75	0.239	0.262	B-DNA
2DPC	2.50	0.155	0.142	B-DNA
2ELG	1.70	0.153	0.168	Z-DNA
2ET0	1.90	0.210	0.240	

2F8W	1.60	0.215	0.217	Z-DNA
2FIH	2.40	0.216	0.196	B-DNA
2FII	1.80	0.186	0.282	B-DNA
2FIJ	1.50	0.195	0.200	A-DNA
2GB9	2.07	0.140	0.171	B-DNA
2GJB	0.98	0.130	0.152	B-DNA
2GPX	1.60	0.207	0.190	A-DNA
2GVR	1.10	0.104	0.112	B-DNA
2GW0	2.00	0.251	0.253	Quadruplex
2GWE	0.85	0.107	0.114	Quadruplex
2GWQ	1.12	0.181	0.196	Quadruplex
2GYX	1.24	0.153	0.168	B-DNA
2H05	0.86	0.117	0.128	A-DNA
2H0N	1.90	0.161	0.154	Z-DNA
2H9S	2.09	0.161	0.196	Other duplex
2HBN	2.20	0.186	0.173	Quadruplex
2HC7	1.40	0.240	0.232	A-DNA
2HRI	2.00	0.228	0.254	Quadruplex
2HTO	1.10	0.156	0.171	Z-DNA
2I2I	2.52	0.168	0.171	B-DNA
2I5A	1.85	0.177	0.199	B-DNA
2NLM	1.37	0.245	0.290	B-DNA
2NSK	1.28	0.183	0.174	A-DNA
2O1I	1.50	0.202	0.207	B-DNA
2O4F	1.82	0.250	0.284	Quadruplex
2OBZ	1.72	0.204	0.232	Z-DNA
2OKS	1.75	0.192	0.238	B-DNA
2ORF	1.65	0.220	0.244	B-DNA
2ORG	2.30	0.255	0.270	B-DNA

2ORH	1.60	0.128	0.146	B-DNA
2P8D	1.90	0.230	0.254	B-DNA
2PKV	2.20	0.234	0.254	A-DNA
2PL4	1.04	0.165	0.212	A-DNA
2PL8	1.60	0.181	0.193	A-DNA
2PLB	1.75	0.197	0.218	A-DNA
2PLO	0.55	0.082	0.081	A-DNA
2QEF	1.86	0.208	0.238	B-DNA
2QEG	1.30	0.152	0.159	B-DNA
2RF3	1.50	0.228	0.235	B-DNA
302D	1.75	0.164	0.176	B-DNA
303D	1.10	0.172	0.187	B-DNA
307D	1.81	0.226	0.224	B-DNA
308D	2.50	0.230	0.269	Other duplex
311D	2.20	0.206	0.244	B-DNA
312D	2.40	0.205	0.217	Z-DNA
313D	1.25	0.197	0.189	Z-DNA
314D	2.00	0.243	0.257	Z-DNA
317D	1.55	0.238	0.248	A-DNA
318D	2.10	0.239	0.260	A-DNA
319D	0.98	0.118	0.143	A-DNA
321D	2.38	0.206	0.232	A-DNA
322D	2.10	0.172	0.168	A-DNA
323D	1.30	0.152	0.165	A-DNA
324D	1.06	0.241	0.252	A-DNA
325D	1.50	0.254	0.281	A-DNA
326D	1.55	0.191	0.200	A-DNA
327D	1.71	0.217	0.236	A-DNA
334D	1.20	0.174	0.190	B-DNA

335D	2.32	0.144	0.164	B-DNA
337D	1.60	0.201	0.219	A-DNA
338D	1.32	0.157	0.192	A-DNA
340D	1.50	0.242	0.260	A-DNA
341D	1.93	0.196	0.212	A-DNA
343D	2.05	0.236	0.250	A-DNA
345D	1.77	0.162	0.182	A-DNA
348D	1.85	0.230	0.288	A-DNA
349D	1.67	0.216	0.209	A-DNA
352D	1.75	0.205	0.225	Quadruplex
355D	2.50	0.203	0.211	B-DNA
358D	2.20	0.192	0.194	B-DNA
360D	1.97	0.237	0.286	B-DNA
362D	1.80	0.216	0.289	Z-DNA
366D	0.90	0.166	0.170	Other duplex
380D	0.95	0.175	0.209	Other duplex
381D	1.66	0.232	0.224	Other duplex
386D	1.45	0.141	0.134	Other duplex
388D	1.30	0.217	0.215	B-DNA
389D	2.56	0.150	0.172	B-DNA
390D	2.40	0.190	0.212	Z-DNA
3AJK	1.85	0.191	0.164	B-DNA
3ANA	1.95	0.194	0.221	A-DNA
3BM0	1.10	0.152	0.162	A-DNA
3BSE	2.20	0.243	0.289	B-DNA
3C1P	1.25	0.142	0.160	
3CCO	2.50	0.180	0.213	Quadruplex
3CDM	1.42	0.216	0.214	Quadruplex
3CE5	1.67	0.194	0.214	Quadruplex

3CO3	1.65	0.203	0.226	B-DNA
3EM2	1.60	0.199	0.215	Quadruplex
3EQW	1.30	0.184	0.218	Quadruplex
3ERU	2.02	0.180	0.193	Quadruplex
3ES0	2.20	0.165	0.212	Quadruplex
3ET8	1.90	0.199	0.191	Quadruplex
3EUI	2.00	0.219	0.277	Quadruplex
3EUM	1.65	0.202	0.221	Quadruplex
3EY2	2.30	0.248	0.262	A-DNA
3EY3	2.31	0.228	0.252	B-DNA
3F8O	1.60	0.222	0.226	Z-DNA
3FL6	2.15	0.225	0.198	B-DNA
3FQB	1.55	0.183	0.197	Z-DNA
3FT6	1.81	0.209	0.202	B-DNA
3FX8	1.70	0.173	0.207	B-DNA
3G2A	2.15	0.197	0.204	Z-DNA
3GCY	1.60	0.220	0.237	Z-DNA
3GDA	2.00	0.231	0.257	Z-DNA
3GGI	1.38	0.174	0.170	B-DNA
3GGK	1.55	0.168	0.180	B-DNA
3GJK	1.65	0.213	0.231	B-DNA
3GJL	2.50	0.190	0.211	B-DNA
3GNK	1.17	0.167	0.173	B-DNA
3GOM	2.30	0.217	0.235	B-DNA
3GOO	1.65	0.160	0.172	B-DNA
3GSJ	2.00	0.201	0.185	B-DNA
3GSK	1.53	0.189	0.190	B-DNA
3HG8	2.20	0.254	0.263	A-DNA
3HGD	1.60	0.252	0.241	A-DNA

3I1D	0.98	0.146	0.154	
3I5E	1.51	0.154	0.159	B-DNA
3I5L	1.64	0.227	0.250	B-DNA
3IFF	1.25	0.154	0.172	A-DNA
3IFI	2.39	0.226	0.197	A-DNA
3IGT	1.60	0.230	0.231	B-DNA
3IJK	1.55	0.225	0.239	A-DNA
3IJN	1.70	0.207	0.234	A-DNA
3IKI	1.82	0.198	0.228	A-DNA
3K18	1.91	0.193	0.194	A-DNA
3KNC	1.70	0.214	0.216	
3KQ8	2.20	0.171	0.168	A-DNA
3L1Q	2.00	0.183	0.207	B-DNA
3LPV	0.95	0.200	0.203	B-DNA
3LTR	1.90	0.164	0.179	A-DNA
3LTU	2.25	0.184	0.262	A-DNA
3MBS	1.05	0.266	0.266	
3N4N	2.04	0.189	0.243	B-DNA
3NP6	1.78	0.207	0.219	B-DNA
3NYP	2.10	0.227	0.229	Quadruplex
3NZ7	1.10	0.207	0.216	Quadruplex
3OIE	2.00	0.226	0.261	B-DNA
3OMJ	1.70	0.205	0.209	B-DNA
3OPI	1.04	0.135	0.136	B-DNA
3P4J	1.43	0.195	0.194	Z-DNA
3PA0	1.60	0.159	0.180	Other duplex
3PBX	1.19	0.155	0.160	B-DNA
3Q5C	2.11	0.244	0.243	B-DNA
3Q61	1.30	0.171	0.200	A-DNA

3QBA	1.15	0.120	0.138	Z-DNA
3QF8	1.70	0.270	0.266	B-DNA
3QRN	1.50	0.244	0.225	B-DNA
3QSC	2.20	0.212	0.179	Quadruplex
3QSF	2.24	0.201	0.216	Quadruplex
3QXR	1.73	0.178	0.197	Quadruplex
3R6R	2.21	0.196	0.224	Quadruplex
3S80	2.10	0.197	0.213	B-DNA
3SC8	1.45	0.145	0.175	Quadruplex
3SD8	1.84	0.195	0.216	A-DNA
3SSF	1.50	0.218	0.234	
3T5E	1.27	0.180	0.194	Quadruplex
3T86	2.10	0.210	0.213	Quadruplex
3T8P	1.70	0.160	0.168	B-DNA
3TCI	2.22	0.204	0.247	Z-DNA
3TVB	2.10	0.226	0.210	Quadruplex
3U05	2.51	0.245	0.268	B-DNA
3U08	2.30	0.193	0.207	B-DNA
3U0U	1.80	0.147	0.161	B-DNA
3U2N	1.08	0.158	0.168	B-DNA
3U38	1.00	0.164	0.176	B-DNA
3U89	1.85	0.231	0.259	B-DNA
3UYA	1.83	0.223	0.235	B-DNA
3UYB	2.01	0.191	0.242	B-DNA
3UYH	2.20	0.205	0.243	Quadruplex
3V06	1.64	0.226	0.282	A-DNA
3V07	2.40	0.192	0.194	A-DNA
3V9D	2.16	0.218	0.217	A-DNA
3WBO	1.79	0.252	0.277	Z-DNA

400D	1.75	0.252	0.279	Z-DNA
401D	1.40	0.180	0.220	A-DNA
403D	1.22	0.187	0.217	B-DNA
423D	2.50	0.227	0.223	B-DNA
427D	1.94	0.200	0.249	Other duplex
428D	1.60	0.215	0.224	B-DNA
431D	2.00	0.201	0.239	B-DNA
432D	2.42	0.181	0.229	B-DNA
436D	0.98	0.108	0.126	B-DNA
440D	1.60	0.189	0.171	A-DNA
442D	2.05	0.155	0.208	B-DNA
443D	1.90	0.221	0.251	B-DNA
444D	1.08	0.258	0.275	B-DNA
447D	1.75	0.221	0.233	B-DNA
448D	1.00	0.156	0.168	B-DNA
449D	1.50	0.198	0.207	B-DNA
453D	1.97	0.253	0.285	B-DNA
455D	2.50	0.174	0.173	B-DNA
458D	2.25	0.171	0.204	B-DNA
460D	2.30	0.187	0.207	B-DNA
461D	1.40	0.175	0.178	B-DNA
463D	1.60	0.204	0.248	B-DNA
467D	1.50	0.201	0.193	Other duplex
473D	1.16	0.143	0.167	Other duplex
476D	1.70	0.233	0.245	B-DNA
477D	1.60	0.252	0.219	B-DNA
478D	1.10	0.143	0.151	B-DNA
479D	2.40	0.188	0.240	
482D	1.50	0.230	0.235	Other duplex

4AGZ	2.00	0.173	0.196	B-DNA
4AH0	2.50	0.194	0.207	B-DNA
4C5X	2.00	0.165	0.197	B-DNA
4C63	1.20	0.265	0.250	B-DNA
4C64	1.34	0.118	0.148	B-DNA
4DA3	2.20	0.212	0.254	Quadruplex
4DWY	0.75	0.136	0.139	Z-DNA
4DX4	2.10	0.198	0.226	A-DNA
4DY8	1.40	0.202	0.206	Z-DNA
4E2R	1.43	0.193	0.202	Z-DNA
4E4O	2.01	0.188	0.200	Z-DNA
4E60	1.71	0.188	0.216	Z-DNA
4E7Y	1.26	0.250	0.266	A-DNA
4E8S	1.60	0.220	0.223	B-DNA
4E8X	1.76	0.239	0.262	B-DNA
4E95	1.38	0.179	0.178	A-DNA
4EZ2	1.72	0.221	0.246	Other duplex
4F2X	1.48	0.218	0.243	B-DNA
4F2Y	1.50	0.183	0.181	A-DNA
4F3U	1.40	0.168	0.175	B-DNA
4F4N	2.22	0.186	0.231	A-DNA
4F8G	1.99	0.236	0.255	A-DNA
4F8I	2.00	0.212	0.233	A-DNA
4FP6	1.65	0.247	0.244	
4FS5	2.00	0.235	0.256	Z-DNA
4FS6	2.02	0.176	0.180	Z-DNA
4FXM	1.71	0.242	0.261	Quadruplex
4G0F	1.13	0.170	0.183	Quadruplex
4GJU	1.90	0.220	0.218	B-DNA

4GLC	1.88	0.253	0.262	B-DNA
4GLG	1.60	0.227	0.241	B-DNA
4GLH	1.15	0.145	0.152	B-DNA
4GQD	2.25	0.222	0.284	Other duplex
4GRE	1.40	0.207	0.216	Other duplex
4GS2	1.70	0.259	0.272	Other duplex
4GSG	1.65	0.224	0.252	Other duplex
4GSI	2.25	0.188	0.179	Other duplex
4H29	1.90	0.161	0.163	Quadruplex
4H5A	1.76	0.196	0.176	B-DNA
4HIF	2.31	0.182	0.185	Z-DNA
4HIG	2.25	0.202	0.228	Z-DNA
4HLI	1.70	0.249	0.265	B-DNA
4HQI	2.10	0.235	0.255	B-DNA
4I1G	1.72	0.189	0.207	A-DNA
4I9V	1.60	0.201	0.203	B-DNA
4IJ0	1.55	0.166	0.161	B-DNA
4IZQ	1.60	0.203	0.237	A-DNA
4KW0	2.30	0.226	0.267	B-DNA
4L25	1.24	0.214	0.211	Other duplex
4L26	1.90	0.177	0.155	Other duplex
4LTF	2.00	0.200	0.252	Other duplex
4LTH	1.90	0.218	0.229	Other duplex
4LTI	1.45	0.208	0.230	Other duplex
4LTJ	1.10	0.246	0.241	Other duplex
4LTK	2.20	0.247	0.285	Other duplex
4LTL	2.00	0.204	0.193	Other duplex
4LY2	1.70	0.196	0.211	Other duplex
4M3I	1.51	0.138	0.142	Other duplex

4M3V	0.96	0.110	0.111	B-DNA
4MGW	1.10	0.172	0.165	B-DNA
4MKW	2.24	0.200	0.218	B-DNA
4MS5	2.38	0.219	0.272	A-DNA
4O5W	0.89	0.147	0.162	B-DNA
4O5X	2.15	0.228	0.277	B-DNA
4O5Y	2.18	0.221	0.234	B-DNA
4O5Z	1.46	0.191	0.189	B-DNA
4OCB	2.25	0.238	0.248	Z-DNA
4OCD	1.56	0.247	0.263	B-DNA
4OKL	1.40	0.173	0.168	A-DNA
4WO2	2.40	0.198	0.208	Quadruplex
9DNA	1.50	0.175	0.182	A-DNA

Table S3 Summary of peaks in Z-DNA crystal structures

Most popular(P212121) crystal form

a=17.8, b=31.2, c=44.3, alpha=90.0, beta=90.0, gamma=90.0

PDB ID	Resolution	Chain+Residue ID	Residue name	Assigned conformation in PDB	Peak intensity for ZI/ZII#
1D39	1.26	A3	DC	ZI	3.36
1D39	1.26	A5	DC	ZII	
1D39	1.26	B9	DC	ZII	
1D39	1.26	B11	DC	RestZ(ZI-like)	8.16
1D41	1.37	A3	DU	ZI	
1D41	1.37	A5	5CM	ZII	3.07
1D41	1.37	B9	DU	ZI	
1D41	1.37	B11	5CM	ZI	
1D76	1.30	A3	DU	ZI	3.58
1D76	1.30	A5	DC	ZII	3.47
1D76	1.30	B9	DU	ZI	5.69
1D76	1.30	B11	DC	ZI	5.02
1DJ6	1.00	A3	DC	ZI	
1DJ6	1.00	A5	DC	ZII	5.20
1DJ6	1.00	B9	DC	ZI	
1DJ6	1.00	B11	DC	ZI	
1DN5	1.40	A3	CBR	ZI	3.85
1DN5	1.40	A5	CBR	ZI	3.60
1DN5	1.40	B9	CBR	ZI	3.57
1DN5	1.40	B11	CBR	ZI	3.28 (2nd)
1ICK	0.95	A3	DC	ZI	4.96
1ICK	0.95	A5	DC	ZII	
1ICK	0.95	B13	DC	ZI	
1ICK	0.95	B15	DC	ZI	4.54
1VRO	1.10	A3	DC	ZI	3.65 (2nd)
1VRO	1.10	A5	DC	ZI	3.73
1VRO	1.10	B109	DC	ZI	5.63
1VRO	1.10	B111	DC	ZII	3.85
2ELG	1.00	A3	DC	ZI	6.95
2ELG	1.00	A5	DC	ZII	
2ELG	1.00	B9	DC	ZI	
2ELG	1.00	B11	DC	ZI	3.65
2OBZ	1.10	A3	DC	ZI	3.72
2OBZ	1.10	A5	BRU	ZII	4.40

2OBZ	1.10	B9	DC	ZI	6.23
2OBZ	1.10	B11	BRU	ZI	4.46
3P4J	0.55	A3	DC	ZI	
3P4J	0.55	A5	DC	ZII	
3P4J	0.55	B9	DC	ZI	
3P4J	0.55	B11	DC	ZI	
3WBO	0.98	A3	DC	ZI/ RestZ(ZII-like)	-
3WBO	0.98	A5	DC	ZI/ZII	-
3WBO	0.98	B9	DC	ZI/ZII	-
3WBO	0.98	B11	DC	ZI	4.00
4HIF	0.85	A3	DC	ZI	
4HIF	0.85	A5	DC	ZII	
4HIF	0.85	B9	DC	ZI	
4HIF	0.85	B11	DC	ZII	
4HIG	0.75	A3	DC	ZI	3.91
4HIG	0.75	A5	DC	ZII	
4HIG	0.75	B9	DC	ZI	3.78
4HIG	0.75	B11	DC	ZI	5.73

Another P212121 crystal form

a=19.0, b=30.1, c=43.2, alpha=90.0, beta=90.0, gamma=90.0

PDB ID	Resolution	Chain+Residue ID	Residue name	Assigned conformation in PDB	Peak intensity for ZI/ZII#
1OMK	1.30	A3	DC	ZII	4.95
1OMK	1.30	A5	5IU	ZI	5.07
1OMK	1.30	B9	DC	RestZ(ZI-like)	
1OMK	1.30	B11	5IU	RestZ(ZII-like)	
1WOE	1.50	A3	DC	ZI	
1WOE	1.50	A5	DC	ZI	4.11
1WOE	1.50	B9	DC	ZI/ZII	-
1WOE	1.50	B11	DC	ZI	
2F8W	1.20	A3	DC	ZI	
2F8W	1.20	A5	DT	ZI	
2F8W	1.20	B9	DC	ZII	10.08
2F8W	1.20	B11	DT	ZI	
362D	1.30	A3	DC	ZII	7.78
362D	1.30	A5	DC	ZI	
362D	1.30	B9	DC	ZII	
362D	1.30	B11	DC	ZI/ZII	-

P32 crystal form

a=b=18.6, c=72.7, alpha=90.0, beta=90.0, gamma=120.0

PDB ID	Resolution	Chain+Residue ID	Residue name	Assigned conformation in PDB	Peak intensity for ZI/ZII#
4FS5	1.30	A3	DC	ZII	4.88
4FS5	1.30	A5	DC	RestZ(ZII-like)	4.94
4FS5	1.30	B9	DC	ZII	3.99
4FS5	1.30	B11	DC	ZII	5.38

P3221 crystal form

a=b=18.5, c=71.5, alpha=90.0, beta=90.0, gamma=120.0

PDB ID	Resolution	Chain+Residue ID	Residue name	Assigned conformation in PDB	Peak intensity for ZI/ZII#
4FS6	1.30	A3	DC	ZII	
4FS6	1.30	A5	DC	ZII	

C2 crystal form

a=48.5, b=19.5 c=31.2, alpha=90.0, beta=116.4, gamma=90.0

PDB ID	Resolution	Chain+Residue ID	Residue name	Assigned conformation in PDB	Peak intensity for ZI/ZII#
4OCB	0.75	A3	DC	ZI/ZII	-
4OCB	0.75	A5	DC	ZI/ RestZ(ZII-like)	-
4OCB	0.75	A7	DC	ZII	
4OCB	0.75	A9	DC	ZI/ZII	-
4OCB	0.75	A11	DC	ZI/ZII	-

Z-DNA structure with copper-mediated base pair P21 crystal form

a=25.3, b=34.4 c=31.1, alpha=90.0, beta=101.1, gamma=90.0

PDB ID	Resolution	Chain+Residue ID	Residue name	Assigned conformation in PDB	Peak intensity for ZI/ZII#
1JES	1.50	A3	DC	ZII	3.5
1JES	1.50	A5	DPY	ZI	
1JES	1.50	A7	DT	ZII	3.56
1JES	1.50	A9	DC	RestZ(ZI-like)	4.03
1JES	1.50	A11	DC	ZI	
1JES	1.50	B15	DC	ZII	3.01
1JES	1.50	B17	DPY	ZI	
1JES	1.50	B19	DT	ZII	
1JES	1.50	B21	DC	RestZ(ZI-like)	3.27
1JES	1.50	B23	DC	ZI/ZII	-

B/Z mixed structure P21 crystal form

a=27.4, b=39.3 c=30.6, alpha=90.0, beta=98.8, gamma=90.0

PDB ID	Resolution	Chain+Residue ID	Residue name	Assigned conformation in PDB	Peak intensity for ZI/ZII#
4L25	1.10	A3	DC	ZI	
4L25	1.10	A9	DC	ZII	4.10
4L25	1.10	A11	DC	ZI	3.75
4L25	1.10	B3	DC	ZI	
4L25	1.10	B9	DC	ZII	4.00
4L25	1.10	B11	DC	ZI/ZII	-
4L26	1.40	A3	CBR	ZI	
4L26	1.40	A9	DC	ZII	
4L26	1.40	A11	DC	ZI	4.81
4L26	1.40	B3	CBR	ZI	
4L26	1.40	B9	DC	ZII	
4L26	1.40	B11	DC	ZI/ZII	-

Peaks were extracted from the m|Fo|-D|Fc| maps at 1.5 Å resolution. Peak intensity for ZI/ZII indicates the intensity of a peak found in sub-region A for ZI and sub-region C for ZII, respectively. Most peaks listed in the table are highest peaks at a distance within 2.2 Å from the P atoms. If the peak is the second highest one, (2nd) is added after the value of the peak intensity.

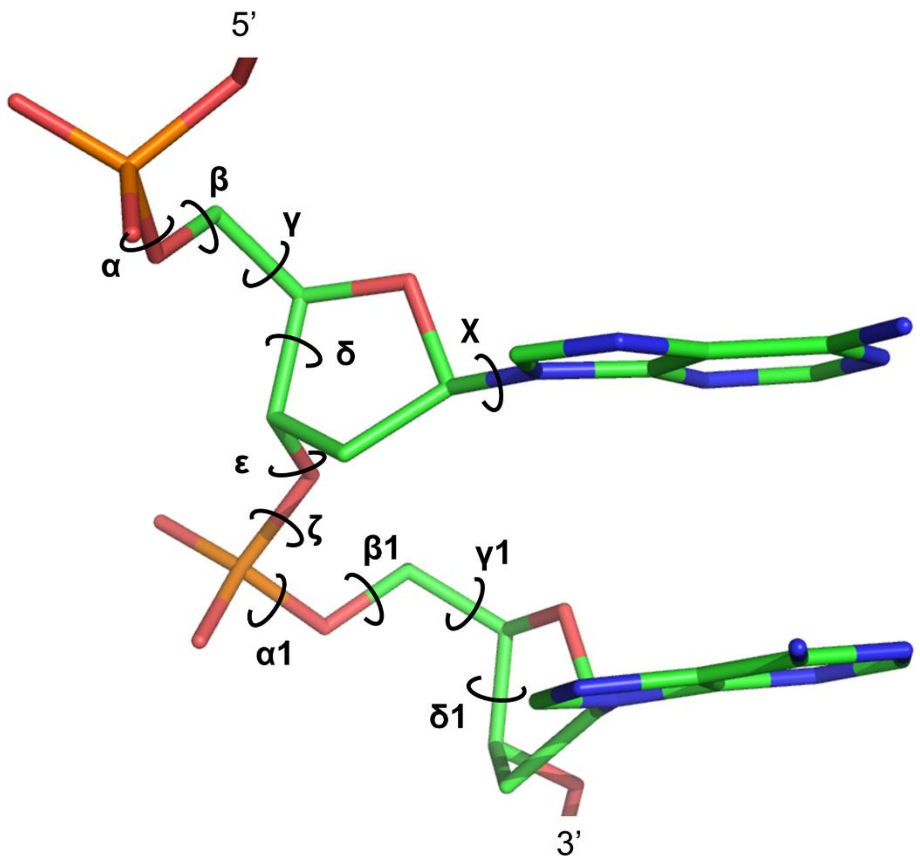

Figure S1 Definition of torsion angles of DNA backbone. This figure illustrates α, β, γ, ε, ζ and χ in a residue, and α1, β1, γ1 and δ1 in the next residue. The canonical B-DNA for this figure was generated by fiber program in X3DNA.

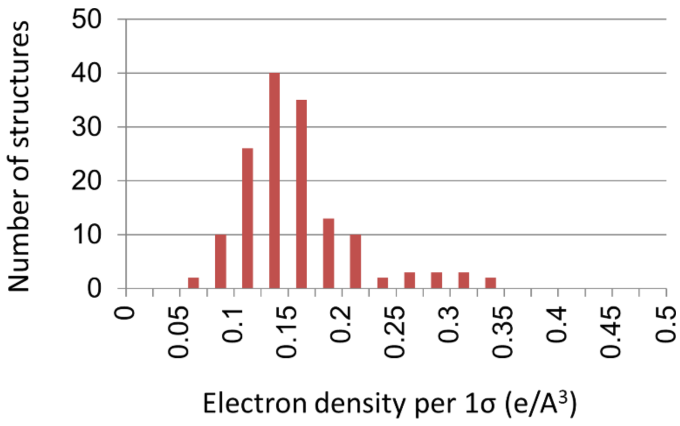

Figure S2 Distribution of the electron densities per 1σ (e/A^3). The median is 0.146 and the standard deviation is 0.051.

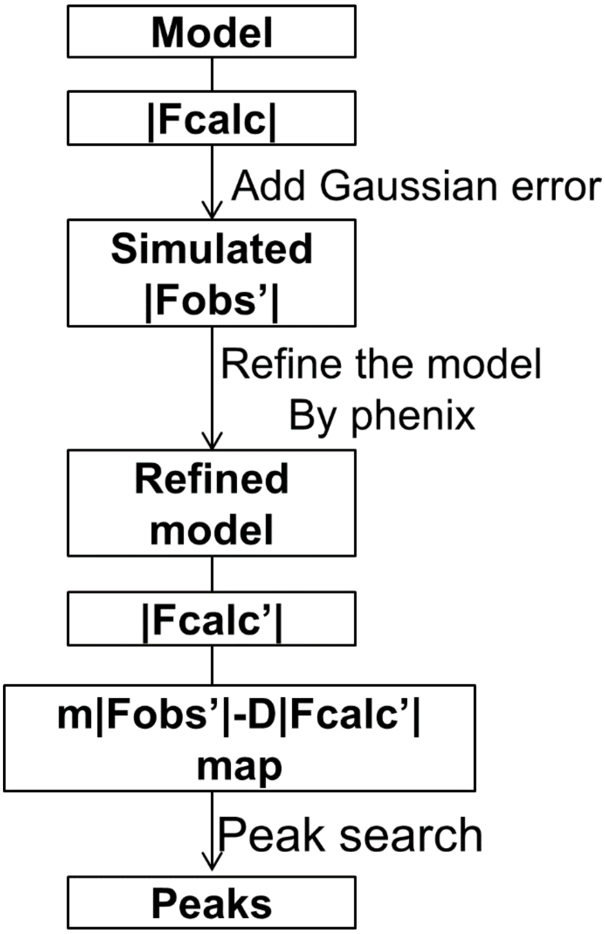

Figure S3 Flowchart of simulation to determine a threshold for the peak picking in the m|Fo|-D|Fc| maps.

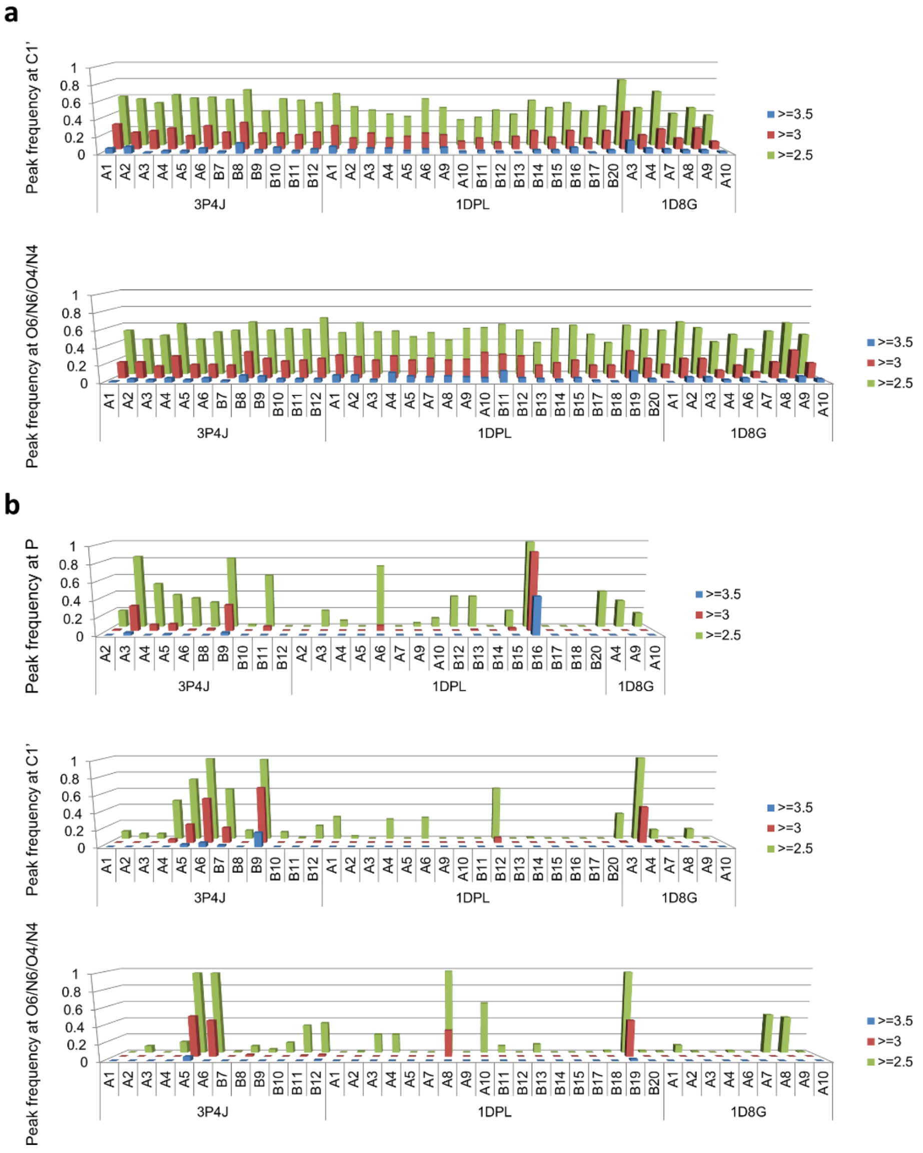

Figure S4 Frequency of peaks produced by noises in the m|Fo|-D|Fc| map. (a) In the simulation using FoFc error, peak frequencies of individual C1' atoms (upper panel) and 6th hetero atoms in purine or 4th hetero atoms in pyrimidine (lower panel) are shown. (b) In the simulation using sigmaF error, peak frequencies of individual P atom (upper panel), C1' atoms (middle panel) and 6th hetero atoms in purine or 4th hetero atoms in pyrimidine (lower panel) are shown. Atoms without alternative locations were used to draw figures.

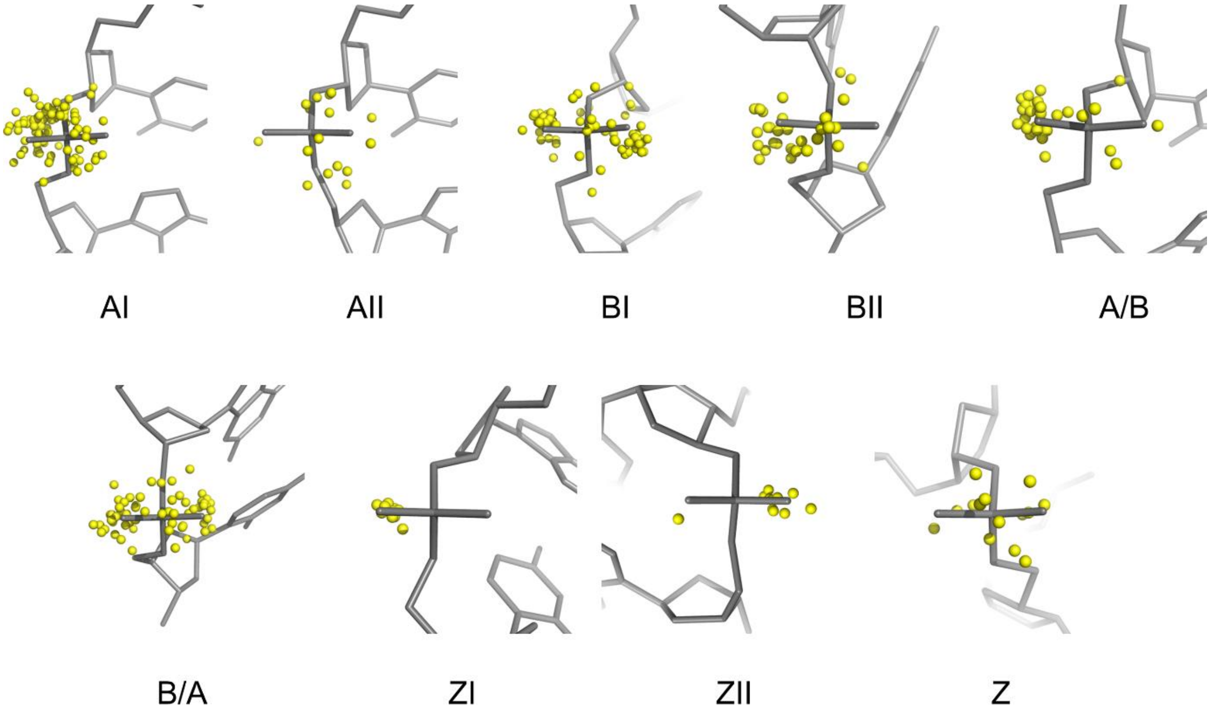

Figure S5 Peaks in 9 types of dinucleotides. Yellow spheres indicate peak locations. Each dinucleotide is superimposed on a representative structure of each conformer (grey). The representative structures were 3IFI [A6-A7] for AI, 3EY2 [A102-A103] for AII, 1FQ2 [A1-A2] for BI, 4I9V [A10-A11] for BII, 460D [A3-A4] for A/B, 4I9V [B22-B23] for B/A, 3P4J [B10-B11] for ZI, 3P4J [A4-A5] for ZII, and 4OCB [A9-A10] for Z (letters in blankets indicate chain IDs and residue IDs). C3’, C4’ and O3’ in the residue and P, OP1, OP2, C5’ and O5’ in the next residue were used for superposition. Only dinucleotides containing DA, DT, DG, or DC were used in both steps to draw these figures. When several peaks were found at the location of an atom, the strongest peak was used to draw figures.

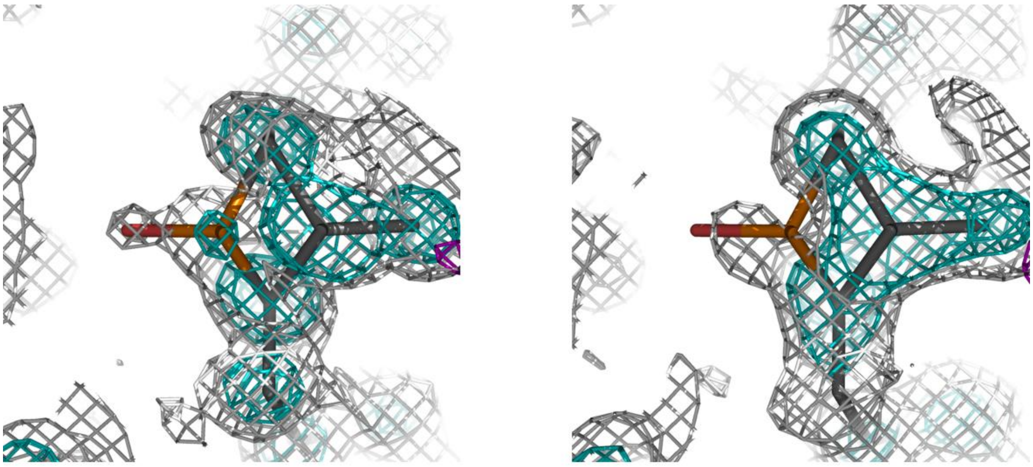

Resolution: 0.98Å Resolution: 1.5Å

Resolution (Å)	without ZI/ZII assignment		ZI/ZII assignment		
	R_{work}	R_{free}	OCC	R_{work}	R_{free}
0.98	0.164	0.180	0.16	0.161	0.179
1.5	0.166	0.205	0.12	0.167	0.204

Figure S6 Example of remodeling of ZI/ZII transitions based on m|Fo|-D|Fc| peaks. We remodeled a Z-DNA structure which was previously solved by one of the authors using a conventional crystallographic analysis method (PDBid 3WBO: Chatake, 2013). We identified potential transitions (4.0 σ peak in 1.5Å resolution m|Fo|-D|Fc| map) in the phosphate at [B10-B11]. The alternative conformation was generated by superimposing these residues on the structure of 4OCB [A8-A9] which contains ZI/ZII multiple conformations. Initial occupancy was set as 0.15 for the added conformation. Then, the coordinate, B-factor and occupancy were refined using phenix. The same procedure was applied for the structure without considering alternative conformation. From the map at 0.98 Å resolution, alternative conformation seems to exist, although R-factor did not significantly change. At 1.5 Å resolution, we could not clearly observe electron density for the alternative conformation. This structure was determined at a low salt concentration. Alternative conformations corresponding to ZI/ZII for the other three of the four GpC steps in the structure have been assigned. Taken together, GpC steps are highly likely to be in equilibrium of ZI/ZII transition at low salt concentrations. Similar results were obtained for 1ICK [A2-A3] (5.0σ) and 1ICK [B14-B15] (4.5σ) of which structure was solved at 0.95 Å resolutions (data not shown).

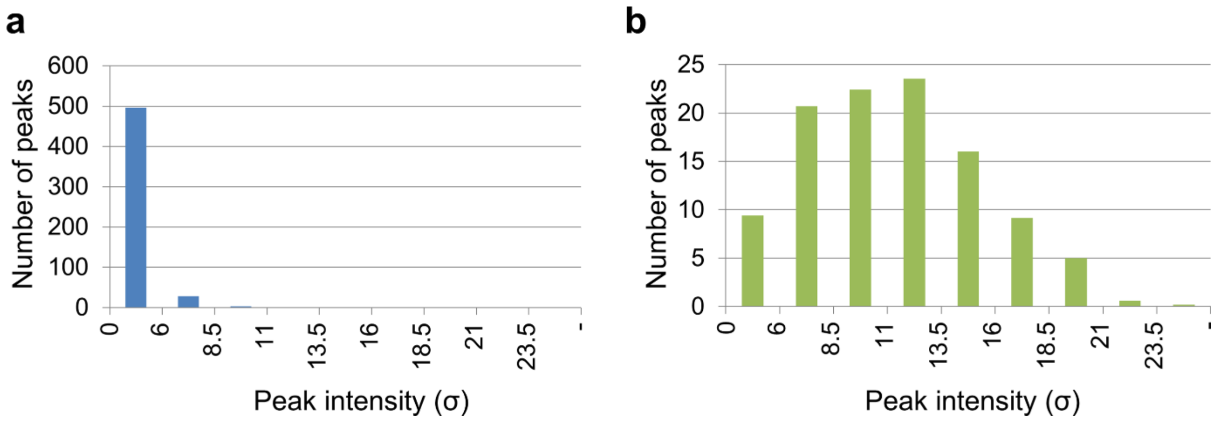

Figure S7 Distribution of peak intensities in the m|Fo|-D|Fc| maps. (a) Peaks at a distance within 2.2 Å from P atoms considered in the present study. When several peaks were found at the location of an atom, the strongest peak was used to draw figures. (b) Distribution of peaks that appeared by removing one of phosphates that were assigned multiple conformations. Atoms removed were one of P, OP1, OP2, and O5’ in the residue and O3’ in the descending residue. The m|Fo|-D|Fc| maps were calculated at a 1.5 Å resolution.

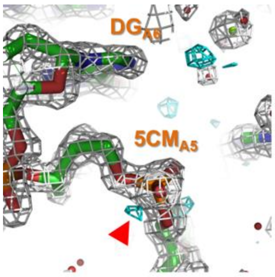

1D41

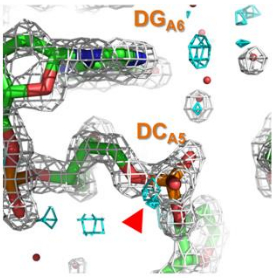

1D76

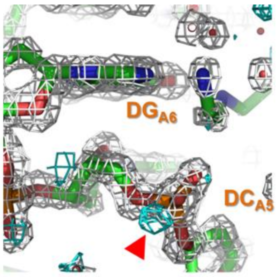

1DJ6

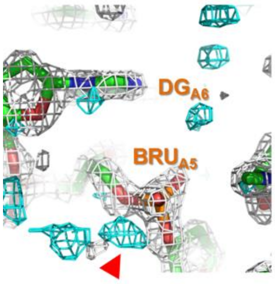

2OBZ

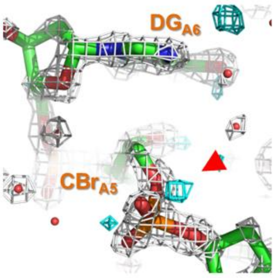

1DN5

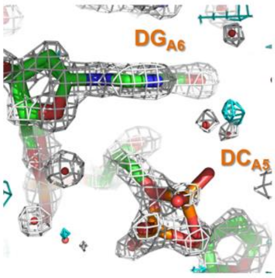

3WBO

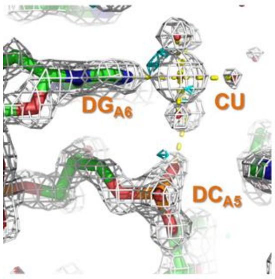

1D39

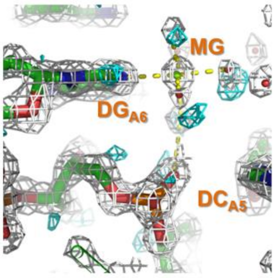

1ICK

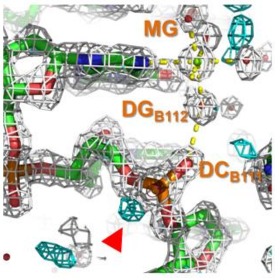

1VRO

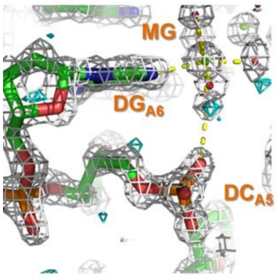

2ELG

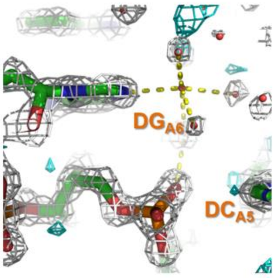

3P4J

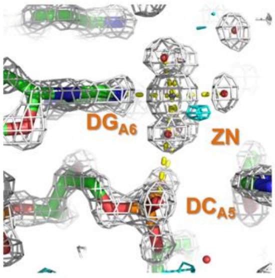

4HIF

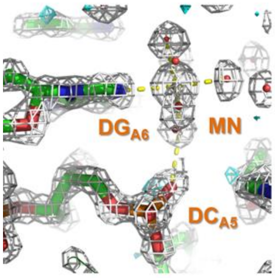

4HIG

Figure S8 Electron density maps in cation/polyamine binding sites. The 2m|Fo|-D|Fc| maps are shown in grey, and the m|Fo|-D|Fc| maps are shown in cyan. The contour levels of 2m|Fo|-D|Fc| and m|Fo|-D|Fc| maps are 1 σ and 2.75 σ, respectively. Peaks in the m|Fo|-D|Fc| maps potentially corresponding to ZI/ZII are highlighted by red arrows. Yellow dotted lines indicate H-bonds or coordination bonds. Residue names were shown in orange, followed by Chain ID and residue ID as subscripts. The resolution used to draw these maps was 1.5 Å.
